# Assessment of a Prediction Model for Antidepressant Treatment Stability Using Supervised Topic Models

**DOI:** 10.1001/jamanetworkopen.2020.5308

**Published:** 2020-05-20

**Authors:** Michael C. Hughes, Melanie F. Pradier, Andrew Slavin Ross, Thomas H. McCoy, Roy H. Perlis, Finale Doshi-Velez

**Affiliations:** 1 Department of Computer Science, Tufts University, Medford, Massachusetts; 2 John A. Paulson School of Engineering and Applied Sciences, Cambridge, Massachusetts; 3 Center for Quantitative Health, Massachusetts General Hospital, Boston; 4 Harvard Medical School, Boston, Massachusetts

## Abstract

**Importance:**

In the absence of readily assessed and clinically validated predictors of treatment response, pharmacologic management of major depressive disorder often relies on trial and error.

**Objective:**

To assess a model using electronic health records to identify predictors of treatment response in patients with major depressive disorder.

**Design, Setting, and Participants:**

This retrospective cohort study included data from 81 630 adults with a coded diagnosis of major depressive disorder from 2 academic medical centers in Boston, Massachusetts, including outpatient primary and specialty care clinics from December 1, 1997, to December 31, 2017. Data were analyzed from January 1, 2018, to March 15, 2020.

**Exposures:**

Treatment with at least 1 of 11 standard antidepressants.

**Main Outcomes and Measures:**

Stable treatment response, intended as a proxy for treatment effectiveness, defined as continued prescription of an antidepressant for 90 days. Supervised topic models were used to extract 10 interpretable covariates from coded clinical data for stability prediction. With use of data from 1 hospital system (site A), generalized linear models and ensembles of decision trees were trained to predict stability outcomes from topic features that summarize patient history. Held-out patients from site A and individuals from a second hospital system (site B) were evaluated.

**Results:**

Among the 81 630 adults (56 340 women [69%]; mean [SD] age, 48.46 [14.75] years; range, 18.0-80.0 years), 55 303 reached a stable response to their treatment regimen during follow-up. For held-out patients from site A, the mean area under the receiver operating characteristic curve (AUC) for discrimination of the general stability outcome was 0.627 (95% CI, 0.615-0.639) for the supervised topic model with 10 covariates. In evaluation of site B, the AUC was 0.619 (95% CI, 0.610-0.627). Building models to predict stability specific to a particular drug did not improve prediction of general stability even when using a harder-to-interpret ensemble classifier and 9256 coded covariates (specific AUC, 0.647; 95% CI, 0.635-0.658; general AUC, 0.661; 95% CI, 0.648-0.672). Topics coherently captured clinical concepts associated with treatment response.

**Conclusions and Relevance:**

The findings suggest that coded clinical data available in electronic health records may facilitate prediction of general treatment response but not response to specific medications. Although greater discrimination is likely required for clinical application, the results provide a transparent baseline for such studies.

## Key Points

**Question:**

To what degree can coded clinical data from electronic health records be used to predict achievement of a stable antidepressant regimen in patients with major depressive disorder?

**Findings:**

In this cohort study of 81 630 adults, 55 303 were identified as having reached an antidepressant treatment regimen that was stable, meaning a clinician elected to continue the same prescription for at least 90 days. Treatment-specific models performed no better than general treatment outcome models in predicting stable antidepressant treatment regimens.

**Meaning:**

The findings suggest that coded clinical data may facilitate prediction of antidepressant treatment outcomes, but medication-specific models do not outperform general response prediction models.

## Teaser

This cohort study assesses medication-specific treatment models vs general treatment models for predicting stable response to antidepressant treatment among patients with major depressive disorder.

## Introduction

Meta-analysis suggests that newer antidepressants are on average similar in efficacy and overall tolerability,^1^ a finding further supported by a small number of effectiveness studies.^2,3,4^ However, these group averages obscure a wide amount of interindividual variability; even before the advent of precision or personalized medicine, the literature^5^ addressed potential predictors of antidepressant treatment outcome aimed at identifying individuals who are more or less likely to benefit. For example, symptom-defined subtypes were investigated initially as predictors of tricyclic antidepressant or monoamine oxidase inhibitor response, then as predictors of selective serotonin reuptake inhibitor response.^6,7,8^ More recently, instead of clinical subtypes, efforts have focused on deriving constellations of symptoms more associated with response^9,10,11^ or on incorporating additional survey measures.^12^ Beyond clinical factors, numerous studies^13,14^ examined incorporation of biomarkers, most notably (and notoriously) the dexamethasone suppression test.

A key challenge in all of these studies^6,7,8,9,10,11,12^ has been the paucity of head-to-head antidepressant studies distinguishing factors associated with poor outcomes overall from factors associated with poor outcomes specific to a given medication is often difficult. Traditional tests of interaction compound this problem because they are best powered for opposing associations (ie, markers associated with better outcome in 1 group and poorer outcome in another), when in reality, this may not comport with biologic characteristics. Furthermore, even in head-to-head studies,^1,15,16^ there are rarely replication cohorts to follow up initial associations.

In other contexts, electronic health record (EHR) or administrative data sets have been used to assess clinical outcomes, providing sufficiently large real-world cohorts to allow identification and validation of predictors.^17,18,19^ They may offer the further advantage of operating on data already readily available at the point of care, such that clinical adoption does not require the use of new rating scales or measures. In the present study, we sought to apply widely available EHR data to assess the extent to which general (ie, nonspecific) predictors of antidepressant response can be identified and whether treatment-specific predictors can be identified and applied to a precision medicine approach to antidepressant prescribing.

In so doing, we also investigated a potential solution to the lack of interpretability, which is a central problem in analysis of large clinical data sets and machine learning for big data in general.^20,21,22^ Although optimized predictions may be useful, the inability to understand what drives these predictions may impede efforts to validate and disseminate them in clinical settings. Moreover, the reliance on individual clinical data points may limit portability if health systems use different procedure or diagnostic codes to reflect the same underlying concepts. Here, we applied a recently developed supervised topic modeling approach^23^ that yields simple predictors based on groups of features that retain discrimination and facilitate interpretability.

## Methods

### Study Design

For this cohort study, we used an in silico cohort drawn from EHRs to examine the association between coded EHRs available at time of medication prescription for standard antidepressants and subsequent longitudinal outcomes of stable treatment with that medication. The Partners HealthCare institutional review board approved the study protocol, waiving the requirement for informed consent since only deidentified data were used and no human persons contact was required. This study followed the Strengthening the Reporting of Observational Studies in Epidemiology (STROBE) reporting guideline.

The study cohort included individuals with at least 1 diagnosis of major depressive disorder (*International Classification of Diseases, Ninth Revision* [*ICD-9*] diagnosis codes 296.2x and 296.3x) or depressive disorder not otherwise specified (311) who received psychiatric care between December 1, 1997, and December 31, 2017, across the inpatient and outpatient networks of 2 large academic medical centers (sites A and B) in New England. Patients were excluded if age was younger than 18 years or older than 80 years, if the total observation period was less than 90 days, or if there were fewer than 3 total documented visits (of any type, psychiatric or otherwise) in the EHR.

We extracted deidentified patient-level data using the i2b2 server software (i2b2 Foundation Inc).^24^ Available patient data included sociodemographic information (age, sex, and race/ethnicity), all diagnostic and procedural codes, and all inpatient and outpatient medication prescriptions.

After applying inclusion criteria (eFigure 1 in the Supplement), a total of 51 048 patients from site A were included and randomly assigned to training (25524 [50%]), validation (12762 [25%]), and test (12762 [25%]) subsets. A total of 26 176 patients from site B composed an external validation set.

### Outcome Definition

Recognizing that traditional clinical trial outcomes such as response and remission are difficult to define reliably for all individuals using solely coded clinical data,^18^ we instead sought to identify individuals who achieved a period of stable treatment as a proxy for ample clinical benefit and tolerability. We applied a simplifying but face-valid assumption that successful treatments continue uninterrupted over time with repeated prescriptions, whereas unsuccessful treatments are either discontinued or require addition of further medication.^4^

We initially considered 27 possible antidepressants (eTable 1 in the Supplement). We defined a treatment segment as stable if it contained at least 2 prescriptions for the same antidepressants on 2 distinct dates at least 30 days apart, the total duration was at least 90 days, the calculated medication possession ratio (fraction of days in segment during which the patient possessed a valid, nonexpired prescription)^25^ was at least 80%, and the largest gap between adjacent prescription dates in the segment was at most 390 days (eFigure 2 and eFigure 3 and eMethods 1 in the Supplement). Only 11 antidepressants had sufficient use at site A (at least 1000 patients) to be used as targets for stability prediction (eTable 1 in the Supplement).

### Covariate Definition

For each patient, available sociodemographic covariates included sex and race/ethnicity (one-hot categorical) as well as date of the visit and age of the patient (numerical). Additional patient covariates included all available coded billing data (ie, *ICD-9* and *International Statistical Classification of Diseases and Related Health Problems, Tenth Revision* [*ICD-10*] diagnoses, *Current Procedural Terminology* laboratory tests, and procedures) and the identity of all prescribed medications. From this initial set of 36 875 possible codes (ie, code words), we selected 9256 code words that occurred for at least 50 patients at site A. Thus, a count vector of 9256 entries represented a patient’s diagnostic and treatment history.

### Classification Methods for General and Drug-Specific Stability

The primary aim of prediction analysis was to identify patients likely to exhibit general stability while receiving antidepressants. Given the patient’s history up to an evaluation date, evaluate whether the patient will be stable after index prescription of any antidepressant treatment. The secondary aim was to assess whether an individual would exhibit drug-specific stability.

One classifier was trained for the general stability outcome as well as a separate drug-specific classifier for each of the 11 target antidepressants. We considered 2 standard probabilistic classifiers, logistic regression and extremely randomized trees, using the open-source implementations in Scikit Learn.^26^ All classifiers were trained on site A’s training set and had hyperparameters selected using grid search on site A’s validation set to maximize the area under the receiver operating characteristic curve (AUC). Final performance was evaluated on both site A’s testing set and the independent cohort from site B. Final performance was evaluated on both site A’s testing set and the independent cohort from site B (eMethods 2 in the Supplement gives training and evaluation details).

### Supervised Topic Models for General and Drug-Specific Stability Prediction

A challenge in machine learning is maintaining interpretability while maximizing predictive performance. Even after applying the frequency threshold, an input space of 9256 code words limits interpretability and risks model overfitting. We thus reduced this coded data set into groups of cooccurring codes indicative of an underlying concept using probabilistic topic models (eFigure 4 in the Supplement).^27^

We applied a recent technique for training topic models to perform supervised predictions called *prediction-constrained* (PC) topic modeling.^23^ Most topic models summarize the most salient concepts in the data. For example, diseases such as diabetes, chronic kidney disease, and cancer are prevalent in health records and thus will always be discovered as topics. However, it is not clear a priori whether these prominent conditions are relevant to predicting treatment response in major depressive disorder; given the importance of comorbidity, solely rediscovering comorbidity might exclude other features important for prediction. Prediction-constrained topic models address this issue, finding concepts useful for specific prediction tasks rather than summarizing prominent elements. We used PC topic models to provide low-dimensional patient-specific covariates that yield comparable performance to classifiers that use high-dimensional code word covariates more interpretable insights into how elements of the patient history factor into prediction. More details on topic modeling applications to coded clinical data has been published previously.^28,29^

On the basis of prior work,^23^ we applied PC training to fit PC–supervised Latent Dirichlet Allocation topic models to site A’s training set. We selected 10 topics as representing the best trade-off between validation performance and model size. Experimental details for training and hyperparameter selection for topic models are included in eMethods 3 in the Supplement. Links to visualizations of trained topic models are included in eResults 1 in the Supplement. Open-source code is available elsewhere.^30^

### Evaluating Suitability of Models for Medication Prioritization

We further sought to assess how drug-specific models could be used to select medications to prioritize for each patient and compared this with clinical practice. Evaluating such prioritized medications requires certain assumptions because, for most patients, we only observed outcomes with 1 or a few of the 11 possible medications. Given the top 3 suggested medications for a patient, we assigned 1 of 3 categories: not assessable (none of the 3 had known stability outcomes for that patient), assessable and stable (at least 1 of the 3 had a positive outcome), and assessable and nonstable (none of the 3 was stable and at least 1 was nonstable). We then computed across a population the top-3 stability accuracy, which indicates the fraction of assessable patients who would have stable response to treatment. This evaluation represented a biased (because models were not trained to prioritize among medications) but potentially useful proxy for a possible future use of drug-specific models.

### Evaluating Models for Forecasting Needed Medication Changes

We evaluated models of general stability by assessing how well they could forecast the number of medication changes that an individual would require before stability is achieved. For each model, we determined a probability score for each patient in site A’s test set, used this to stratify persons into 4 quartiles, and then reported for each quartile the mean number of medication initiations observed in practice before achieving stability.

### Statistical Analysis

Statistical analysis was conducted between January 1, 2018, and March 15, 2020. We used software written in the Python language, version 2.7 (Python Software Foundation) using open-source packages including NumPy, version 1.11 (NumPy developers) and Scikit-Learn, version 0.18 (Scikit-Learn). To report classification performance measures, we reported means across all 11 target antidepressants on the heldout set as well as CIs computed using the 2.5th and 97.5th percentiles across 5000 bootstrap samples of the heldout test set. We did not perform any significance tests.

## Results

The cohort was composed of 81 630 adults (56 340 women [69%]; mean [SD] age, 48.46 [14.75] years; range, 18.0-80.0 years) across both sites who met the inclusion criteria based on diagnosis and treatment duration (eTable 1 in the Supplement). After exclusion of 4133 patients who lacked any code history before the first visit and thus could not have personalized predictions and 273 persons from site B who had no outcomes for the 11 target antidepressants, 51 048 patients remained from site A (33 961 women [67%]; mean [SD] age, 48.50 [14.90] years) and 26 176 patients remained from site B (19 391 women [74%]; mean [SD] age, 48.96 [14.21] years). The individuals from site A were divided into training, validation, and testing sets, and the individuals from site B were used for external evaluation of models. Sociodemographic characteristics are summarized in eTable 2 in the Supplement, with further descriptive statistics in eFigure 5 in the Supplement.

### Stability Outcome Prevalence and Face Validity

For psychiatrist-treated patients at site A (n = 11 985), we observed that 2642 (22%) never reached stability, 5274 (44%) reached stability with the index prescription, and 4069 (34%) reached stability by the end of the individual’s active care interval (as defined in eMethods 2 in the Supplement). In contrast, for primary care patients at site A (n = 41 658), we observed that 14 208 (34%) never reached stability, 19 867 (48%) reached stability with the index prescription, and 7583 (18%) reached stability by the end of the individual’s active care interval. Overall at site A (n = 53 643), we observed that 16 850 patients (31%) never reached stability, 25 141 (47%) reached stability with the index prescription, and 11 652 (22%) reached stability at the end of the individual’s active care interval (eResults 2 in the Supplement gives additional results for both sites).

### Comparison of Feature Representations at Site A

**Figure 1.**
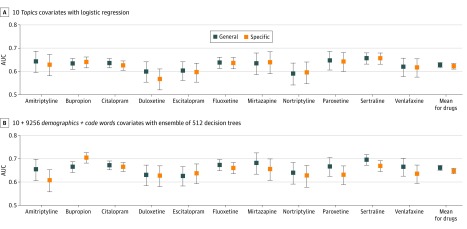
Comparison of General and Drug-Specific Stability Prediction for Proposed and Baseline Covariates Comparison of discriminative ability, as measured by area under the receiver operating characteristic curve (AUC), for general and drug-specific prediction models. A, Models compared use of the proposed 10-dimensional topics covariates with a logistic regression predictor. B, Models compared use of the baseline high-dimensional demographics and words covariates with an ensemble of 512 extremely randomized decision trees. For each of the 11 target antidepressants, an AUC score was obtained for a given model by considering predictions from that model on the subset of the site A test set that included all known outcomes associated with that drug (ignoring data from patients who were never given that drug). To indicate uncertainty in reported AUC values, the evaluation was repeated across 5000 bootstrap samples of each test set and reported error bars indicating 95% CIs for the AUC across these bootstrap samples.

Figure 1 compares general and drug-specific models for 2 possible feature representations: high-dimensional code word count vectors plus demographics and the low-dimensional topics covariates provided by the PC–supervised Latent Dirichlet Allocation topic model. General stability performance was best with demographics and words features and an ensemble of 512 decision trees, achieving a mean AUC of 0.661 (95% CI, 0.648-0.672). When using a simpler logistic regression classifier, the high-dimensional demographics and words features yielded a mean AUC of 0.628 (95% CI, 0.614-0.639). The 10-covariate topic representation captured much of this discriminative capability even when using simple logistic regression, achieving a mean AUC of 0.627 (95% CI, 0.615-0.639). eFigure 6 and eTables 3-6 in the Supplement give comparisons of all feature-classifier combinations at site A.

Figure 1 shows that in contrast to the general stability ensemble model’s mean AUC of 0.661, the drug-specific models achieved a mean AUC of 0.647 (95% CI, 0.635-0.658) when using the same settings: an ensemble of 512 decision trees that used high-dimensional demographics and words features. Using the supervised topic model features and a linear classifier, drug-specific performance on site A reached a mean AUC of 0.627 (95% CI, 0.615-0.639).

### Generalization of Stability Outcome Predictions to Site B

Next, we examined the transferability of models trained on data from site A to separate patients from site B (eTable 2 in the Supplement gives sociodemographic characteristics). Distribution of stability outcomes for site B was similar to that for site A. Among all 27 987 persons, 13 018 (47%) reached stability with the index prescription, 5492 (20%) reached stability by the end of the active care interval, and 9477 (34%) never reached stability.

**Figure 2.**
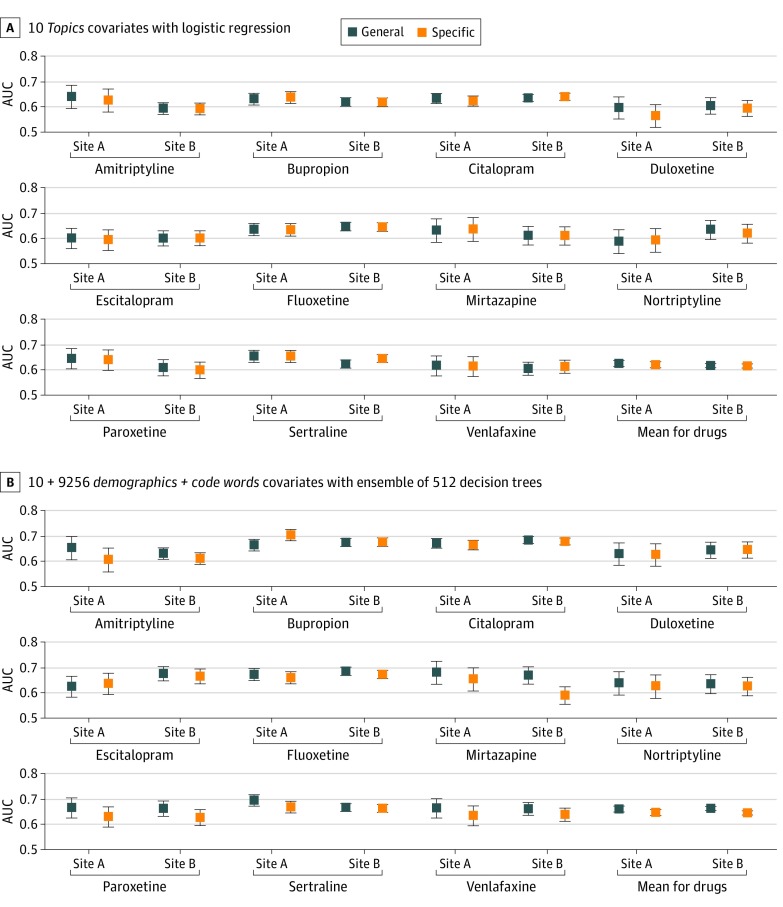
Assessment of Generalization From Site A to Site B for Both General and Drug-Specific Stability Prediction Side-by-side comparison of discriminative ability on the site A and site B testing sets, as measured by area under the receiver operating characteristic curve (AUC), for general and drug-specific prediction models. A, Models use the proposed 10-dimensional topics covariates with a logistic regression predictor. B, Models use the baseline high-dimensional demographics and words covariates with an ensemble of 512 extremely randomized decision trees. For each of the 11 target antidepressants, an AUC score was obtained for a given model by considering predictions from that model on the subset of the site A test set that included all known outcomes associated with that drug (ignoring data from patients who were never given that drug). To indicate uncertainty in reported AUC values, the evaluation was repeated across 5000 bootstrap samples of each test set and reported error bars indicating 95% CIs for the AUC across these bootstrap samples.

Figure 2 shows general stability prediction for both site A and site B, again comparing high-dimensional demographics and words features with the 10-dimensional topic features. Models trained on site A transferred to site B with only modest decay in AUC for both feature representations. Using demographics and words features, the mean AUC was 0.661 (95% CI, 0.648-0.672) for site A and 0.663 (95% CI, 0.654-0.671) for site B. Using the 10-dimensional topic features, the mean AUC was 0.627 (95% CI, 0.615-0.639) for site A and 0.619 (95% CI, 0.610-0.627) for site B. As an alternative evaluation, eFigure 7 in the Supplement plots positive predictive value vs negative predictive value for each model and site.

### Model Interpretability Qualitative Evaluation

We sought to understand which features were important for stability prediction. The Table presents representative topics learned by the proposed 10-topic model for general stability. All topics showed sufficient coherence to enable a qualitative description annotated by one of us (R.H.P.). For example, although both topics 5 and 7 captured routine primary care visits, topic 5 reflected more terms associated with a psychiatric evaluation, suggesting more aggressive intervention or more severe illness. Topic 1 included terms indicative of treatment resistance. Topic 2 captured gynecologic outpatient practice, and topic 4 recorded menopause. The eResults 1 in the Supplement includes hyperlinks to an online visualization tool to explore the important features of all trained models; eFigure 8 in the Supplement shows important features for the demographics and words classifiers.

**Table.** Visualization of Representative Topics From Proposed Supervised Topic Model^a^

Probability	Type^b^	ID	Word
**Topic 5: primary care with some psychiatry, LR coefficient, –1.0**			
0.033	*CPT*	99213	Office visit >15 min
0.024	*CPT*	99214	Office visit >25 min
0.016	*CPT*	99211	Office visit >5 min
0.015	*CPT*	08527	Complete blood count tests
0.010	*CPT*	82565	Creatinine blood test
0.010	*ICD*	78900	Abdominal pain
0.010	*CPT*	85025	Complete blood count tests
0.010	*CPT*	71020	Radiologic examination of chest
0.009	*CPT*	84520	Urea nitrogen laboratory test
0.009	Prescription	42347	Bupropion prescription
0.009	*ICD*	311	Depressive disorder
**Topic 9: back or joint pain, LR coefficient, –0.1**			
0.098	*CPT*	97110	Physical therapy
0.052	*ICD*	7245	Back ache
0.037	*CPT*	97140	Manual therapy
0.033	Prescription	7804	Oxycodone treatment
0.032	*ICD*	7242	Lumbago
0.019	Prescription	214182	Acetaminophen or hydrocodone
0.018	*ICD*	7231	Cervicalgia
0.015	*ICD*	71941	Shoulder pain
0.012	Prescription	25480	Gabapentin
0.012	*ICD*	71947	Ankle or foot pain
0.012	*ICD*	71596	Osteoarthritis of lower leg
**Topic 2: Primary care for younger women, LR coefficient, +0**			
0.034	*CPT*	87591	Test for gonorrhea
0.033	*CPT*	87491	Test for chlamydia
0.020	*CPT*	87070	Bacterial culture
0.017	*CPT*	81025	Urine pregnancy test
0.017	*CPT*	84702	hCG test
0.016	*CPT*	87086	Bacterial culture from urine
0.015	*CPT*	V762	Cervical screening
0.015	*ICD*	462	Acute pharyngitis
0.015	*CPT*	V222	Incidental pregnancy
0.015	*CPT*	76856	Pelvic ultrasonography
0.015	*ICD*	6259	Female genital concern
**Topic 4: primary care for older women, LR coefficient, +0.1**			
0.048	*CPT*	v761	Mammogram
0.038	Prescription	10582	Levothyroxine
0.032	*ICD*	2449	Hypothyroidism
0.025	*ICD*	2724	Hyperlipidemia
0.021	*CPT*	76092	Mammogram
0.020	*CPT*	v762	Cervical screening
0.019	*ICD*	6272	Menopause
0.017	*ICD*	73300	Osteoporosis
0.013	*ICD*	v103	Breast cancer history
0.012	*ICD*	6961	Psoriasis
0.012	*ICD*	78079	Malaise and fatigue
**Topic 1: Treatment resistant major depressive disorder, LR coefficient, +0.3**			
0.138	*ICD*	29630	Major depressive disorder
0.125	*CPT*	90806	Psychotherapy
0.098	*ICD*	90862	Pharmacologic management
0.034	*ICD*	30000	Anxiety
0.032	Prescription	2598	Clonazepam
0.023	*ICD*	29650	Bipolar disorder
0.023	*ICD*	30490	Drug dependency
0.022	*CPT*	90870	Electroconvulsive therapy
0.018	*ICD*	30981	Posttraumatic stress disorder
0.018	*ICD*	2967	Bipolar disorder
0.018	*CPT*	90807	Psychotherapy
0.012	*ICD*	6961	Psoriasis
0.012	*ICD*	78079	Malaise and fatigue
**Topic 7: primary care, LR coefficient +0.7**			
0.048	*CPT*	99213	Office visit >15 min
0.037	*CPT*	99214	Office visit >25 min
0.029	*CPT*	99211	Office visit >5 min
0.021	*CPT*	36415	Blood samples obtained for laboratory test
0.016	*CPT*	85027	Complete blood count
0.016	*CPT*	v700	Routine examination
0.016	Prescription	7646	Omeprazole treatment
0.013	*CPT*	80061	Lipid panel
0.011	*CPT*	90658	Influenza vaccination
0.011	*CPT*	99215	Office visit >40 min
0.011	*CPT*	80053	Metabolomic tests

Abbreviations: *CPT*, *Current Procedural Terminology*; *ICD*, *International Classification of Diseases*; hCG, human chorionic gonadotropin; LR, logistic regression.

^a^ Six learned topics from our proposed Latent Dirichlet Allocation topic model trained to predict general stability that were selected as representative of the 10 total topics learned by the model. Code words with high probability in the same topic were likely to cooccur together in a patient’s record explained by that topic. The top 10 most probable codes are shown. Each topic is labeled with a clinician annotated title (provided post hoc by R.H.P.) and the topic’s index order within the original model. Learned LR coefficients were rounded to the nearest 0.1 for the task of predicting general stability. Large positive coefficients suggest that a patient whose history uses more of that topic will be more stable.

^b^ Each topic is defined by a learned distribution over 9256 possible diagnostic (*ICD*), procedural (*CPT*), and medication-related code words.

### Medication Prioritization vs Clinical Practice

We evaluated the top-3 stability accuracy achieved by models used to prioritize antidepressants for a patient (eTable 7 in the Supplement). When always predicting the same 3 medications most commonly stable in site’s A training set, we measured top-3 stability accuracy to be 0.602 (95% CI, 0.591-0.612; 64.1% of the 12 762 patients in site A’s test set were assessable). For observed clinical practice (in which 1 medication was prescribed in most regimens, but more medications were prescribed in some), the top-3 stability accuracy was 0.602 (95% CI, 0.593-0.611; 99.5% of 12 762 patients assessable). This improved to 0.637 (95% CI, 0.628-0.646; 99.8% of 12 762 patients assessable) if we allowed prescriptions with fewer than 3 medications to be filled up to a total of 3 medications by selecting from the most commonly stable antidepressants. By comparison, the extremely randomized trees model using all demographic and diagnostic code features achieved a top-3 accuracy of 0.622 (95% CI, 0.610-0.634; 47.4% of 12 762 patients assessable). Performance with the topic model was poorer: top-3 accuracy was 0.581 (95% CI, 0.566-0.594; 38.2% of 12 762 patients assessable).

### General Stability Prediction to Forecast Needed Medication Changes

Finally, we assigned all individuals in the test set to a stability risk quartile by their general stability probability score (eTable 8 in the Supplement). For the extremely randomized tree model using all demographics and code words, those in the top quartile had a mean number of additional medication trials of 0.736 (95% CI, 0.688-0.796) beyond the initial prescription at first visit to achieve stability. Those in the bottom quartile required a mean of 1.754 medication trials (95% CI, 1.681-1.843 trials) beyond the initial prescription to achieve stability. By comparison, using the topic model features and logistic regression classifier, the top quartile had a mean number of additional medication trials of 0.864 (95% CI, 0.816-0.918), whereas the bottom quartile has a mean of 1.722 trials (95% CI, 1.647-1.799 trials).

## Discussion

In this analysis of EHRs from more than 81 000 individuals across 2 health systems, we identified machine learning models that predicted achievement of treatment stability, a proxy for effectiveness, based solely on coded clinical data already available instead of incorporating research measures or questionnaires.

The discrimination was modest, with AUCs in the range of 0.60-0.66. However, we were unable to identify any similar published studies in generalizable cohorts, thus we could not make a direct comparison with another method. Whereas an AUC of 0.8 is often seen as a commonly used threshold distinguishing good performance in some studies, others^31,32^ have argued that this makes little sense because the necessary discrimination depends critically on the context in which prediction is applied.

Contrary to our hypothesis, development of treatment-specific predictors instead of general predictors did not meaningfully improve prediction. This may reflect the observation that much of antidepressant response may be considered to be placebo-like or nonspecific. That is, although antidepressants consistently demonstrate superiority to placebo,^1^ placebo response is substantial such that nonspecific predictors may outperform drug-specific ones. This result is consistent with the lack of success of efforts to find treatment-specific pharmacogenomic predictors.^33^ Our results do not preclude the existence of such medication-specific predictors but suggest that other strategies may be required to identify them.

We also presented a framework for understanding the behavior of our drug-specific models if used to guide antidepressant selection, comparing performance with observed clinical practice and with a baseline in which all patients received the most common antidepressants. It bears emphasis that this represented an instance of transfer learning: the models were not trained to recommend antidepressants per se, nor to mimic clinician performance. However, it showed a likely application of these models in practice to personalize treatment selection. We found that the difference between clinician performance and suggesting the one-size-fits-all medications was modest (approximately 3%). Because of the known similarities in efficacy between standard treatments, essentially all of which were derived from a common set of assumptions about monoaminergic neurotransmission, this finding was not surprising. Despite enthusiasm about personalized medicine, the hypothesis that personalization improves outcomes has rarely been rigorously tested to our knowledge. However, the observation that our best models yielded results similar to those of clinicians suggests that clinical performance may not be as out of reach as AUCs alone might indicate.

Our analysis also suggests that general stability prediction may be useful for stratifying patients and understanding personalized chances of stability. We described an approach to estimating the number of treatment trials that may be avoided or saved in which models were applied. The top quartile of predicted stability required about 1 fewer medication trial than the bottom quartile, which suggests that devoting more care resources (eg, more intensive care management or scalable evidence-based therapies) to those in the lower quartiles might be a worthy targeted investment.

Our results also suggest that although topic modeling may not improve prediction compared with high-dimensional representations, it yields readily interpretable concepts relevant to prediction. Electronic health record data are widely acknowledged to be noisy, with codes applied inconsistently even by individual clinicians; in general, using high-dimensional EHR covariates for any study, it is easy to learn predictors that capture site effects or serve as proxies for some other variables. Conversely, the individual coded terms ranked as most important (eTable 6 in the Supplement) were inconsistent between linear and nonlinear models, and many were difficult to align with clinical practice, further illustrating the advantage in interpretability of topic-based models. Our approach, which mapped EHR dimensions into interpretable topics, may allow stakeholders to easily inspect the learned topic features to understand what cooccurring code word features in patient history influence predictions. This property is critical for researchers seeking to understand more complex models and ultimately for clinicians who may use them; nominating treatments without understanding why they are favored is unlikely to be accepted by clinicians accustomed to their own type of personalization.^34^ The transferability of our results to a second health system suggests a further advantage, namely that topics may be more robust to overfitting than individual token-based approaches. In other words, if the goal is to build models that generalize across health systems, supervised topics may help to avoid the tendency of code-based models to fit site-specific use of individual procedure or diagnostic codes.

Some studies^35^ have sought to emphasize a common primary care depression screening tool, such as the Patient Health Questionnaire–9, which characterizes symptom frequency, not severity, and was not designed to measure response. Other studies^18^ have relied on text from narrative clinical notes. However, these approaches may minimize the strengths (availability of large scale, if imperfect, data that correspond to real-world experience) while emphasizing the weaknesses (lack of precision in diagnosis and symptom measurement) in health records. Moreover, they perpetuate the myth that depression symptoms are purely episodic; in reality, such symptoms tend to wax and wane over time for many patients.

In contrast to previous efforts,^18,35^ we used a simple metric to assess stability based on historical prescribing data, assuming that effective and well-tolerated treatments would be continued and ineffective or intolerable medications would be discontinued. We attempted to answer the question, “if I write a prescription today, how likely am I to continue writing it for the next 90 days?”

These results should be considered a starting point; incorporation of additional outcomes and additional clinician- and patient-level factors may improve the quality of assessment. Improving assessment of individual treatment response will require data from multiple modalities. If such estimates are integrated with coded data to form topics, it may be possible to achieve greater discrimination while preserving portability and to understand the key features associated with that discrimination in a way not possible with other machine learning strategies. Once such models emerge, prospective investigation will be needed to assess the extent to which they meaningfully improve outcomes, if at all.

### Limitations

This study has limitations. The outcome that we examined, stability, was markedly different from standard outcomes in clinical trials, such as remission or 50% reduction in symptoms. The standard approach to using EHR data has been to impose a clinical trial–like structure and outcome measures, that is, to extract or impute measures of depression severity.

## Conclusions

The findings suggest that coded clinical data available in EHRs may facilitate prediction of stable treatment response to any antidepressant in general, whereas predictions that are specific to a particular antidepressant perform no better than the general prediction. The findings further suggest that features derived from supervised topic models provide more interpretable insights compared with raw coded features. Although greater discrimination is likely required for clinical application, the results provide a transparent baseline for such studies.

## Notes

Supplement. **eFigure 1.** Flow Diagram Allocating Subjects to Experimental Subsets **eTable 1.** List of 11 Target Antidepressants and All 27 Possible Antidepressants **eFigure 2.** Example Treatment Histories and Stability Outcomes (Simple) **eFigure 3.** Example Treatment Histories and Stability Outcomes (Complex) **eFigure 4.** Illustration of Proposed Topic Model Transformation of EHR Data **eTable 2.** Sociodemographic Summary of Site A and Site B Patients **eFigure 5.** Histograms of Treatment History Statistics by Stability Outcome **eFigure 6.** General Stability AUC Comparison by Feature **eTable 3.** AUC on Site A for General Stability XRT Classifiers **eTable 4.** AUC on Site A for Drug-Specific Stability XRT Classifiers **eTable 5.** AUC on Site A for General Stability LR Classifiers **eTable 6.** AUC on Site B for General Stability XRT Classifiers **eFigure 7.** PPV and NPV Tradeoffs for General Stability Classifiers **eFigure 8.** Important Features for XRT and LR Classifiers **eTable 7.** Top-3 Stability Accuracy Comparison of Models With Clinical Practice **eTable 8.** Number of Medication Changes Needed by Predicted Stability Quartile **eResults 1.** Visualization of Learned Models **eResults 2.** Results: Stability Outcomes for Patients at Site A and Site B **eMethods 1.** Procedures for Study Design, Outcome Definition, and Prediction Task Formulation **eMethods 2.** Procedures for Classifier Training and Hyperparameter Selection **eMethods 3.** Procedures for Topic Model Training and Hyperparameter Selection **eReferences.**
