# Supplementary material for: Assessment of a Prediction Model for Antidepressant Treatment Stability Using Supervised Topic Models
Source: JAMA Netw Open. 2020 May 20;3(5):e205308. doi: 10.1001/jamanetworkopen.2020.5308 (PMC7240354; doi:10.1001/jamanetworkopen.2020.5308)
Supplement: Supplement. — eFigure 1. Flow Diagram Allocating Subjects to Experimental Subsets eTable 1. List of 11 Target Antidepressants and All 27 Possible Antidepressants eFigure 2. Example Treatment Histories and Stability Outcomes (Simple) eFigure 3. Example Treatment Histories and Stability Outcomes (Complex) eFigure 4. Illustration of Proposed Topic Model Transformation of EHR Data eTable 2. Sociodemographic Summary of Site A and Site B Patients eFigure 5. Histograms of Treatment History Statistics by Stability Outcome eFigure 6. General Stability AUC Comparison by Feature eTable 3. AUC on Site A for General Stability XRT Classifiers eTable 4. AUC on Site A for Drug-Specific Stability XRT Classifiers eTable 5. AUC on Site A for General Stability LR Classifiers eTable 6. AUC on Site B for General Stability XRT Classifiers eFigure 7. PPV and NPV Tradeoffs for General Stability Classifiers eFigure 8. Important Features for XRT and LR Classifiers eTable 7. Top-3 Stability Accuracy Comparison of Models With Clinical Practice eTable 8. Number of Medication Changes Needed by Predicted Stability Quartile eResults 1. Visualization of Learned Models eResults 2. Results: Stability Outcomes for Patients at Site A and Site B eMethods 1. Procedures for Study Design, Outcome Definition, and Prediction Task Formulation eMethods 2. Procedures for Classifier Training and Hyperparameter Selection eMethods 3. Procedures for Topic Model Training and Hyperparameter Selection eReferences. [file jamanetwopen-3-e205308-s001.pdf]

Supplementary Online Content

Hughes MC, Pradier MF, Ross AS, McCoy Jr TH, Perlis RH, Doshi-Velez F. Assessment of a prediction model for antidepressant treatment stability using supervised topic models. *JAMA Netw Open*. 2020;3(5):e205308. doi:10.1001/jamanetworkopen.2020.5308

eFigure 1. Flow Diagram Allocating Subjects to Experimental Subsets
eTable 1. List of 11 Target Antidepressants and All 27 Possible Antidepressants
eFigure 2. Example Treatment Histories and Stability Outcomes (Simple)
eFigure 3. Example Treatment Histories and Stability Outcomes (Complex)
eFigure 4. Illustration of Proposed Topic Model Transformation of EHR Data
eTable 2. Sociodemographic Summary of Site A and Site B Patients
eFigure 5. Histograms of Treatment History Statistics by Stability Outcome
eFigure 6. General Stability AUC Comparison by Feature
eTable 3. AUC on Site A for General Stability XRT Classifiers
eTable 4. AUC on Site A for Drug-Specific Stability XRT Classifiers
eTable 5. AUC on Site A for General Stability LR Classifiers
eTable 6. AUC on Site B for General Stability XRT Classifiers
eFigure 7. PPV and NPV Tradeoffs for General Stability Classifiers
eFigure 8. Important Features for XRT and LR Classifiers
eTable 7. Top-3 Stability Accuracy Comparison of Models With Clinical Practice
eTable 8. Number of Medication Changes Needed by Predicted Stability Quartile
eResults 1. Visualization of Learned Models
eResults 2. Results: Stability Outcomes for Patients at Site A and Site B
eMethods 1. Procedures for Study Design, Outcome Definition, and Prediction Task Formulation
eMethods 2. Procedures for Classifier Training and Hyperparameter Selection
eMethods 3. Procedures for Topic Model Training and Hyperparameter Selection
eReferences.

This supplementary material has been provided by the authors to give readers additional information about their work.

© 2020 Hughes MC et al. *JAMA Network Open.*

eFigure 1. Flow Diagram Allocating Subjects to Experimental Subsets

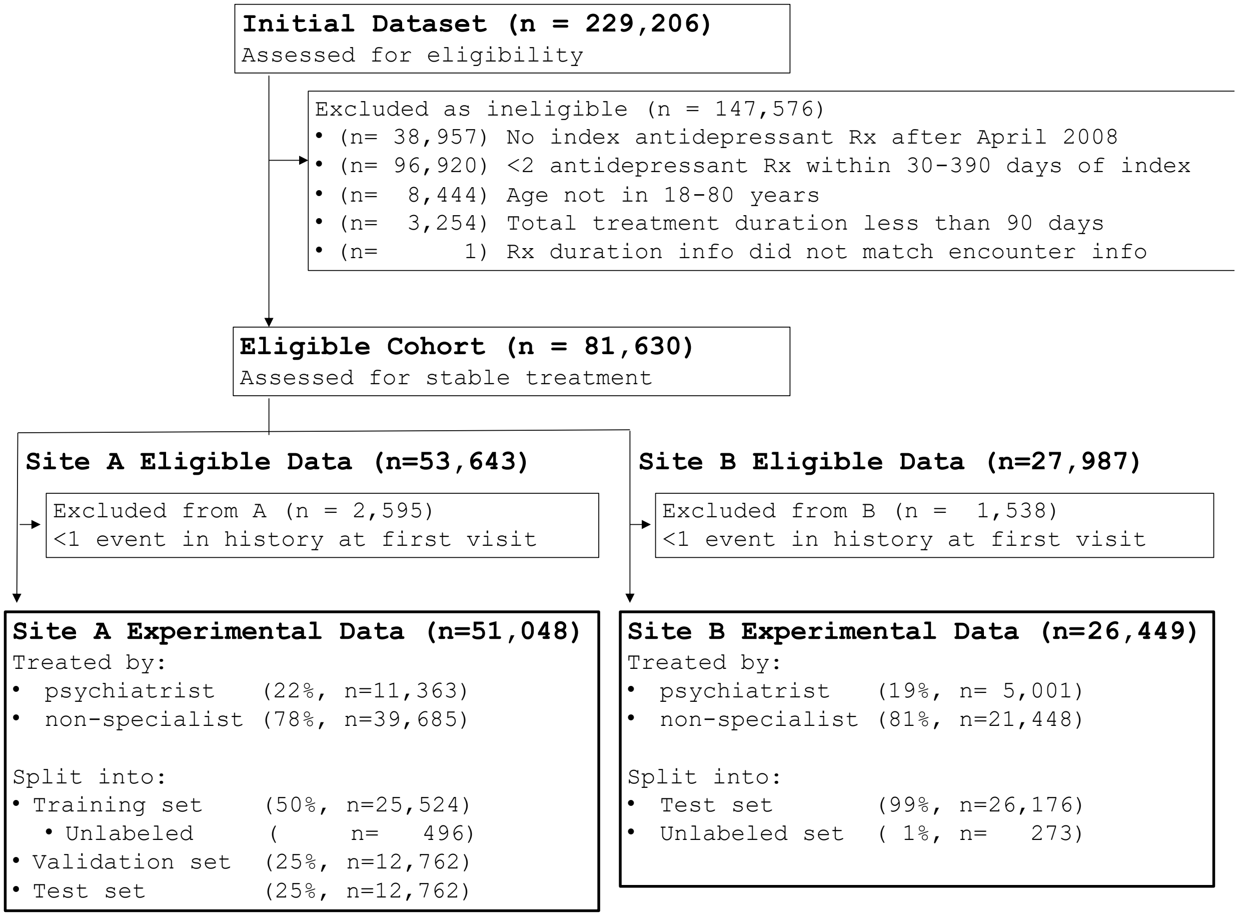

Supplemental eFigure 1: Diagram of how initial preliminary patient population was filtered to obtain a targeted cohort for training and evaluating predictors of antidepressant treatment stability. To determine eligibility, we applied the index visit criteria and sufficient follow-up criteria described in “Procedures for Study Design, Outcome Definition, and Prediction Task Formulation”. The final experimental datasets used only the 11 most common antidepressants, listed in Supplemental eTable 1, as drug-specific outcomes to be predicted and evaluated. Some subjects had no experience with any of these drugs in the active care interval and were thus “unlabeled”. We included such subjects from Site A when training our topic models (which can leverage unlabeled data), but not when training direct history-to-stability classifiers. We did not use unlabeled subjects from Site B when evaluating classifiers, because no target outcome was known. Training, validation, and test sets were split in a way that balanced the empirical fraction of stability outcomes across subsets for each target antidepressant.

© 2020 Hughes MC et al. *JAMA Network Open.*

eTable 1. List of 11 Target Antidepressants and all 27 Possible Antidepressants

drug_name	total count	stable count	stable frac
citalopram	14820	8543	0.576
bupropion	13020	5728	0.440
sertraline	10619	6066	0.571
fluoxetine	9625	5128	0.533
venlafaxine	4549	2043	0.449
escitalopram	4196	1938	0.462
duloxetine	4045	1627	0.402
mirtazapine	3976	1439	0.362
paroxetine	3495	1719	0.492
nortriptyline	3254	1674	0.514
amitriptyline	3087	1387	0.449

drug_name	total count
fluvoxamine	390
desvenlafaxine	283
desipramine	232
imipramine	197
clomipramine	189
vilazodone	123
tranylcypromine	92
nefazodone	82
vortioxetine	70
phenelzine	62
protriptyline	14
amoxapine	13
trimipramine	13
levomilnacipran	9
isocarboxazid	6
maprotiline	6

Supplemental eTable 1: *Top:* List of the 11 target antidepressants chosen to assess stability predictions in this study. Total counts indicate how many distinct patients in the Site A Eligible Dataset were ever prescribed each drug. All target drugs satisfy a minimum frequency criterion: prescribed to over 1000 patients at Site A. Stable count indicates how many distinct patients for which this drug satisfied our stability outcome definition. *Bottom*: Our study considered 27 possible antidepressants; here we show the 16 that did not have sufficient data to build and assess stability prediction models.

© 2020 Hughes MC et al. *JAMA Network Open.*

eFigure 2. Example Treatment Histories and Stability Outcomes (Simple)

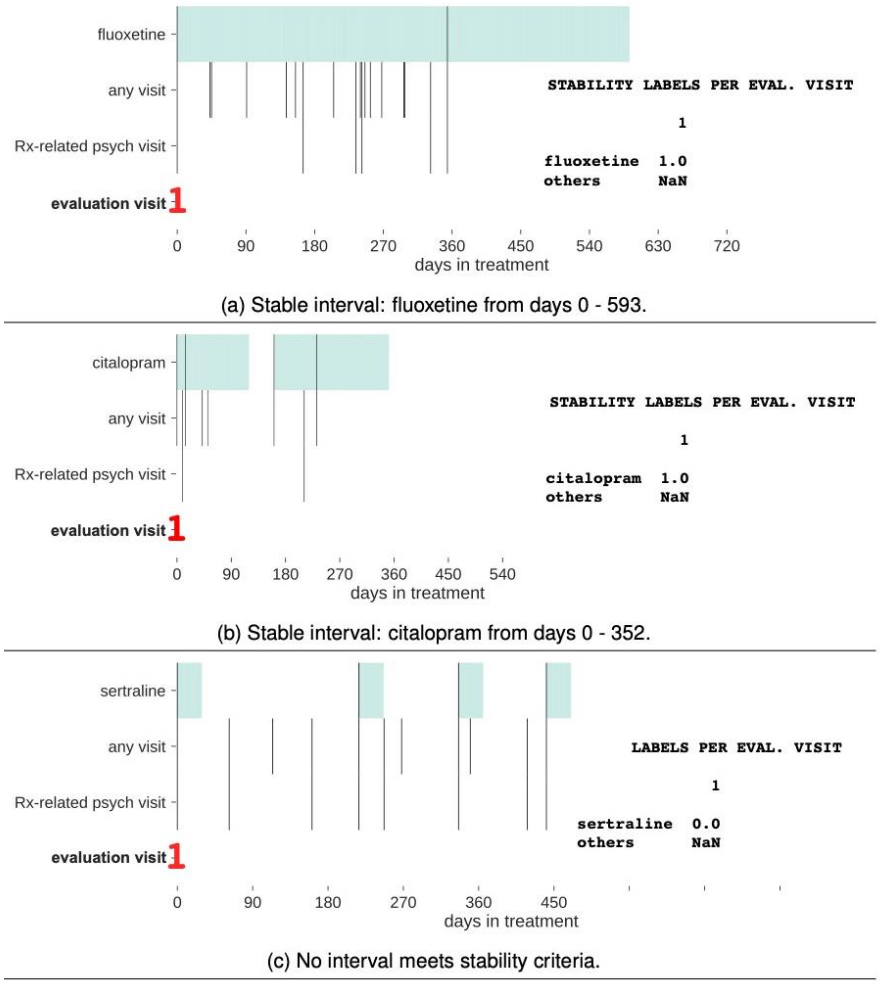

Supplemental eFigure 2: Visualizations of antidepressant treatment history for representative patients (a), (b), and (c) selected to represent simple treatment histories. *Top rows of each panel*: Prescription events for target drugs shown with black lines, while the fill color indicates the duration of each prescription. Duration is computed for each prescription based on recorded dispensed quantities and stop dates. *Middle rows of each panel*: Black lines indicate days when any medical visit occurred (“any visit”), or when psychiatric specialty visits occurred (“Rx-related psych visit”). *Bottom rows of each panel*: Red numbers indicate events where a new segment of antidepressant treatment begins. These are moments when the physician thinks a change is necessary, but may not know which drug will be effective. These dates could be eligible as evaluation dates for our stability prediction models. Subject in panel (a) was stable in their first prescription with fluoxetine (which was renewed around 360 days later). Subject in panel (b) received several citalopram prescriptions over a year and met the stability criteria. Subject in panel (c) received several sertraline prescriptions, but their durations were all too short and lacked renewal/follow-up to meet stability criteria.

© 2020 Hughes MC et al. *JAMA Network Open.*

eFigure 3. Example Treatment Histories and Stability Outcomes (Complex)

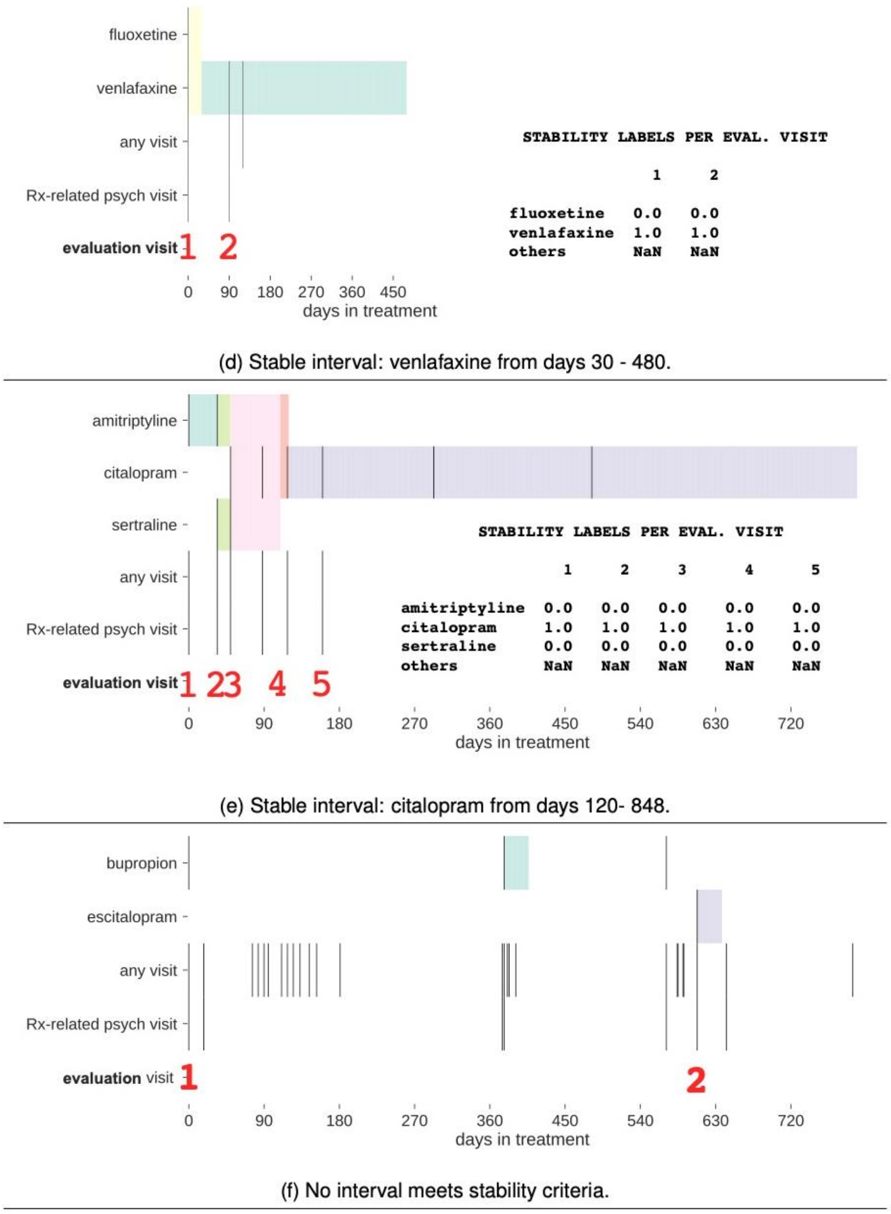

(d) Stable interval: venlafaxine from days 30 - 480.

(e) Stable interval: citalopram from days 120- 848.

(f) No interval meets stability criteria.

Supplemental eFigure 3: Visualizations of antidepressant treatment history for representative patients (d), (e), and (f) with more complex histories. See caption of Supplemental Figure 3 for interpretation of each panel's rows. Subject in panel (d) first tried fluoxetine and venlafaxine (unstable), before switching to just venlafaxine (stable). Subject in panel (e) Tried many different treatments before eventual stability with citalopram. Subject in panel (f) had two 1-day prescriptions for bupropion (each with an enforced stop date), as well as a brief later attempt with escitalopram. None of (f)'s treatment segments met the criteria for stability.

© 2020 Hughes MC et al. *JAMA Network Open.*

eFigure 4. Illustration of Proposed Topic Model Transformation of EHR Data

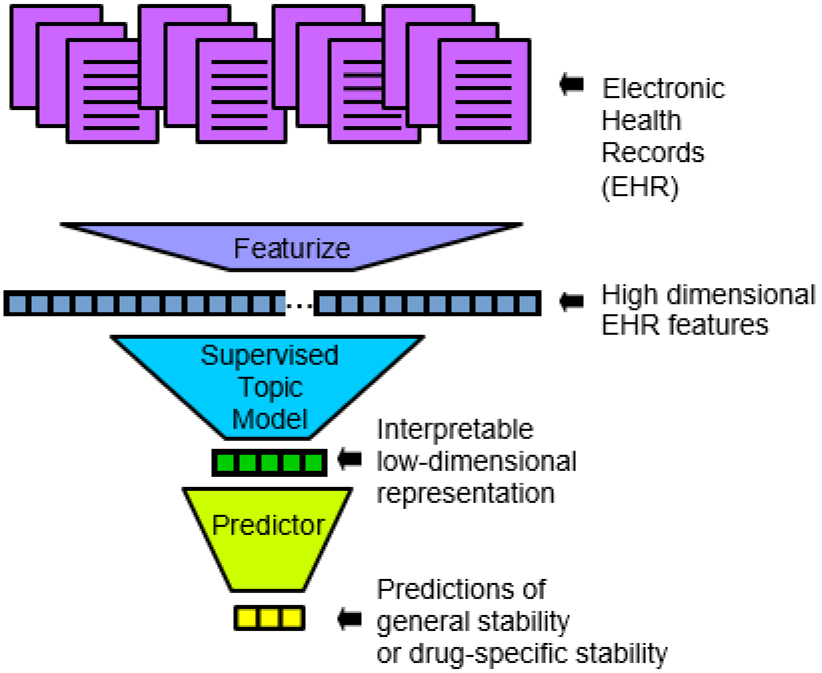

Supplemental eFigure 4: Diagram illustration of how patient data turns into feature vector used to predict stability outcomes. Electronic health records (EHR) are first processed into a high-dimensional count vector of 9256 possible features (diagnoses, procedures, and medication prescriptions). Next, we compress these into a more interpretable, low-dimensional form using supervised topic models. We infer a per-visit low-dimensional feature vector and use it to make predictions about whether each drug is likely to be part of the patient's stable treatment drug list.

© 2020 Hughes MC et al. *JAMA Network Open.*

eTable 2. Sociodemographic Summary of Site A and Site B Patients

Site A Experimental Dataset				Site B Experimental Dataset		
	n	Proportion			n	Proportion
Total	51048	1.000		Total	26176	1.000
Married/partner	22271	0.436		Married/partner	11632	0.444
Female	33961	0.665		Female	19391	0.741
White	42313	0.829		White	17893	0.684
Black	2050	0.040		Black	2623	0.100
Other	6685	0.131		Other	5660	0.216
	mean	sd			mean	sd
ACCI	2.60	3.40		ACCI	2.51	3.16
Age	48.50	14.90		Age	48.96	14.21
Fact count	1136.82	1319.05		Fact count	1226.68	1294.59

Supplemental eTable 2: Demographics of all patients from two studied medical centers (Site A and Site B) who satisfy all required criteria to be evaluated by our prediction algorithms. *Top:* counts and frequencies of marital or long-term partner status, sex identities, and racial identities for all patients at each site. Bottom: Descriptive statistics of patient ages, age-adjusted Charlson comorbidity index score (ACCI), and fact count (number of total ICD9 / ICD10 / CPT / medication codeword events across entire electronic health record).

© 2020 Hughes MC et al. *JAMA Network Open.*

eFigure 5. Histograms of Treatment History Statistics by Stability Outcome

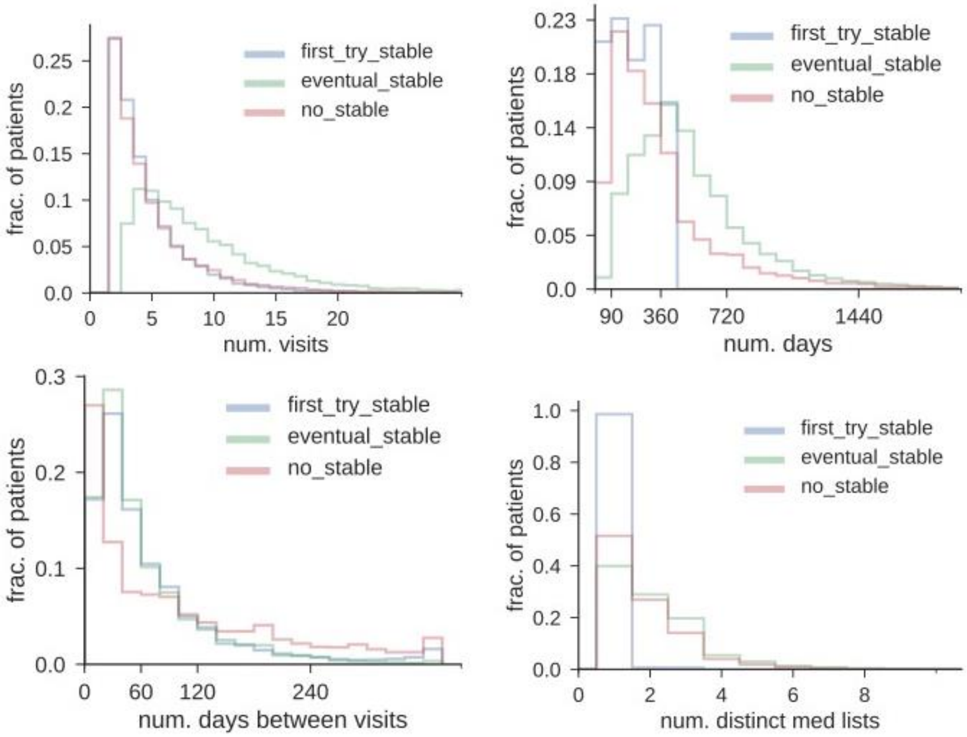

Supplemental eFigure 5: Distribution plots showing the empirical distributions of several key variables for three mutually-exclusive groups of patients from Site A: those with stable treatment on the first prescribed antidepressant, those that eventually reached stable treatment, and those that never achieved stable treatment. All plots are made using data from each patient's active treatment interval (as defined in Supplemental eMethods 1), which lasts at least 90 days, contains no gaps longer than 13 months, and ends after the first successful treatment interval or the end of the record. Top left: Histogram of total number of treatment visits (distinct dates when an antidepressant prescription occurred) during the active treatment period. Top right: Histogram of total duration (in days) of the active treatment period. Bottom left: Histogram of median interval (in days) between treatment visits. By definition, the longest allowed interval length is 13 months (390 days). Bottom right: Histogram of total number of distinct treatments (distinct combinations of the 11 target drugs). By definition, first-try stable patients use only one treatment.

© 2020 Hughes MC et al. *JAMA Network Open.*

eFigure 6. General Stability AUC Comparison by Feature

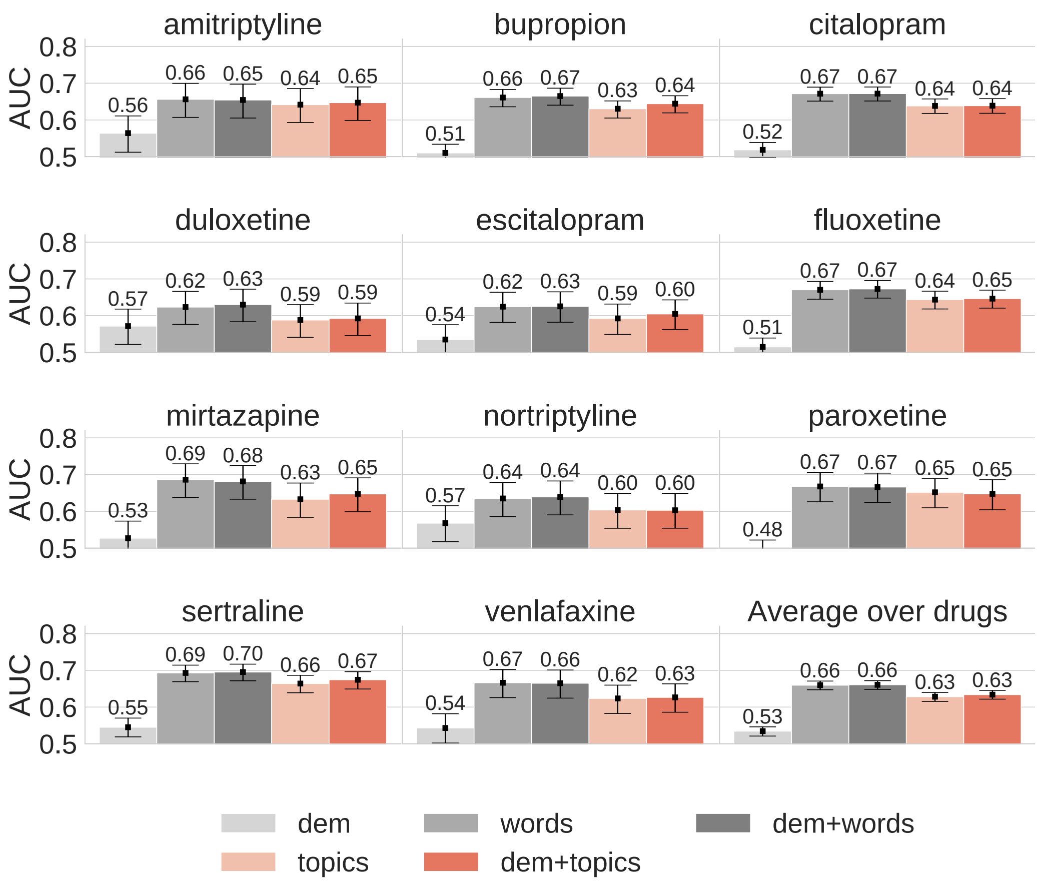

Supplemental eFigure 6: Comparison of different representations of patient history for general stability prediction. Each color represents a different feature representation: “dem” is basic demographic information, “words” is the 9256-dimensional sparse histograms of codeword counts found in the EHR history, and “topics” is the 10-dimensional covariate vector extracted using our learned PC-sLDA topic model (reducing each observed 9256-dimensional count vector to 10 dimensions). Combinations that use our learned topic features are shaded red, baselines without topics are shaded gray. For each feature combination, we trained an ensemble of 512 extremely randomized decision trees on the Site A training set, with hyperparameters tuned on the Site A validation set to maximize the area under the ROC curve (AUC) score. To indicate uncertainty, we show error bars with 95% confidence intervals of the AUC (i.e. scores at 2.5 and 97.5 percentiles) computed from 5000 bootstrap samples of the test set.

© 2020 Hughes MC et al. *JAMA Network Open.*

eTable 3. AUC on Site A for General Stability XRT Classifiers

	dem	words	dem+words	topics
amitriptyline	0.564 (0.512, 0.611)	0.656 (0.607, 0.699)	0.654 (0.605, 0.697)	0.642 (0.593, 0.685)
bupropion	0.510 (0.484, 0.534)	0.661 (0.636, 0.683)	0.665 (0.640, 0.686)	0.630 (0.605, 0.652)
citalopram	0.519 (0.498, 0.539)	0.671 (0.651, 0.689)	0.672 (0.652, 0.690)	0.638 (0.618, 0.657)
duloxetine	0.571 (0.522, 0.618)	0.623 (0.576, 0.666)	0.630 (0.584, 0.672)	0.588 (0.541, 0.630)
escitalopram	0.535 (0.489, 0.575)	0.624 (0.582, 0.664)	0.625 (0.582, 0.665)	0.592 (0.549, 0.632)
fluoxetine	0.515 (0.487, 0.539)	0.670 (0.645, 0.693)	0.673 (0.648, 0.696)	0.644 (0.618, 0.667)
mirtazapine	0.527 (0.474, 0.573)	0.686 (0.638, 0.729)	0.681 (0.633, 0.724)	0.633 (0.584, 0.677)
nortriptyline	0.568 (0.517, 0.615)	0.635 (0.586, 0.679)	0.639 (0.590, 0.683)	0.604 (0.554, 0.649)
paroxetine	0.482 (0.439, 0.522)	0.668 (0.626, 0.706)	0.666 (0.624, 0.704)	0.652 (0.610, 0.690)
sertraline	0.545 (0.519, 0.570)	0.693 (0.669, 0.714)	0.695 (0.671, 0.717)	0.664 (0.639, 0.686)
venlafaxine	0.543 (0.502, 0.582)	0.666 (0.626, 0.702)	0.665 (0.624, 0.701)	0.624 (0.583, 0.660)
avg	0.534 (0.521, 0.546)	0.660 (0.647, 0.671)	0.661 (0.648, 0.672)	0.628 (0.615, 0.640)

Supplemental eTable 3: AUC performance for different feature representations evaluated on the Site A test set. Using *extreme randomized trees (XRT)* classifiers trained to predict *general* stability.

© 2020 Hughes MC et al. *JAMA Network Open.*

eTable 4. AUC on Site A for Drug-Specific Stability XRT Classifiers

	dem	words	dem+words	topics
amitriptyline	0.541 (0.489, 0.587)	0.607 (0.557, 0.653)	0.607 (0.557, 0.652)	0.624 (0.573, 0.667)
bupropion	0.540 (0.515, 0.563)	0.698 (0.674, 0.719)	0.704 (0.681, 0.725)	0.642 (0.616, 0.663)
citalopram	0.526 (0.505, 0.546)	0.659 (0.639, 0.677)	0.665 (0.645, 0.683)	0.626 (0.605, 0.645)
duloxetine	0.576 (0.528, 0.619)	0.617 (0.569, 0.659)	0.627 (0.580, 0.669)	0.581 (0.532, 0.623)
escitalopram	0.617 (0.575, 0.656)	0.625 (0.580, 0.665)	0.637 (0.593, 0.677)	0.587 (0.543, 0.626)
fluoxetine	0.490 (0.462, 0.515)	0.667 (0.641, 0.690)	0.660 (0.635, 0.683)	0.638 (0.612, 0.662)
mirtazapine	0.547 (0.494, 0.594)	0.683 (0.636, 0.727)	0.655 (0.607, 0.698)	0.649 (0.599, 0.694)
nortriptyline	0.575 (0.525, 0.622)	0.634 (0.585, 0.676)	0.628 (0.577, 0.670)	0.600 (0.551, 0.645)
paroxetine	0.493 (0.450, 0.533)	0.634 (0.593, 0.672)	0.631 (0.588, 0.668)	0.645 (0.602, 0.683)
sertraline	0.543 (0.517, 0.568)	0.664 (0.640, 0.687)	0.669 (0.644, 0.691)	0.656 (0.631, 0.679)
venlafaxine	0.561 (0.519, 0.601)	0.638 (0.597, 0.676)	0.635 (0.593, 0.672)	0.622 (0.581, 0.660)
avg	0.546 (0.534, 0.558)	0.648 (0.635, 0.659)	0.647 (0.635, 0.658)	0.624 (0.612, 0.636)

Supplemental eTable 4: AUC performance for different feature representations evaluated on the Site A test set. Using *extreme random trees (XRT)* classifiers trained to predict *specific* stability.

© 2020 Hughes MC et al. *JAMA Network Open.*

eTable 5. AUC on Site A for General Stability LR Classifiers

	dem	words	dem+words	topics
amitriptyline	0.530 (0.477, 0.577)	0.640 (0.590, 0.684)	0.639 (0.589, 0.684)	0.643 (0.595, 0.687)
bupropion	0.524 (0.498, 0.547)	0.632 (0.607, 0.654)	0.633 (0.608, 0.655)	0.634 (0.609, 0.655)
citalopram	0.506 (0.485, 0.526)	0.639 (0.619, 0.658)	0.640 (0.620, 0.659)	0.636 (0.615, 0.655)
duloxetine	0.589 (0.539, 0.633)	0.599 (0.548, 0.644)	0.600 (0.550, 0.645)	0.599 (0.553, 0.641)
escitalopram	0.558 (0.513, 0.600)	0.590 (0.546, 0.628)	0.590 (0.545, 0.628)	0.603 (0.561, 0.641)
fluoxetine	0.492 (0.465, 0.516)	0.634 (0.609, 0.658)	0.636 (0.611, 0.660)	0.638 (0.612, 0.661)
mirtazapine	0.573 (0.521, 0.620)	0.631 (0.579, 0.677)	0.632 (0.580, 0.678)	0.635 (0.586, 0.679)
nortriptyline	0.575 (0.526, 0.621)	0.628 (0.580, 0.672)	0.628 (0.579, 0.672)	0.590 (0.541, 0.636)
paroxetine	0.502 (0.458, 0.543)	0.619 (0.577, 0.658)	0.620 (0.578, 0.658)	0.647 (0.606, 0.686)
sertraline	0.535 (0.509, 0.560)	0.657 (0.632, 0.680)	0.658 (0.633, 0.681)	0.657 (0.631, 0.679)
venlafaxine	0.545 (0.503, 0.583)	0.629 (0.588, 0.665)	0.628 (0.587, 0.665)	0.620 (0.578, 0.657)
avg	0.539 (0.526, 0.551)	0.627 (0.614, 0.639)	0.628 (0.614, 0.639)	0.627 (0.615, 0.639)

Supplemental eTable 5: AUC performance for different feature representations evaluated on the Site A test set. Using *logistic regression (LR)* classifiers trained to predict *general* stability.

© 2020 Hughes MC et al. *JAMA Network Open.*

Supplemental eTable 6: AUC on Site B for General Stability XRT Classifiers

	dem	words	dem+words	topics
amitriptyline	0.511 (0.487, 0.535)	0.606 (0.581, 0.629)	0.608 (0.583, 0.630)	0.596 (0.571, 0.618)
bupropion	0.494 (0.474, 0.512)	0.614 (0.596, 0.631)	0.617 (0.599, 0.635)	0.622 (0.604, 0.638)
citalopram	0.485 (0.470, 0.499)	0.636 (0.621, 0.649)	0.638 (0.623, 0.651)	0.637 (0.623, 0.651)
duloxetine	0.540 (0.505, 0.573)	0.605 (0.570, 0.637)	0.608 (0.574, 0.640)	0.607 (0.572, 0.638)
escitalopram	0.563 (0.530, 0.592)	0.628 (0.597, 0.656)	0.627 (0.596, 0.655)	0.603 (0.571, 0.632)
fluoxetine	0.506 (0.487, 0.523)	0.656 (0.638, 0.672)	0.658 (0.640, 0.674)	0.649 (0.631, 0.665)
mirtazapine	0.570 (0.531, 0.604)	0.659 (0.621, 0.693)	0.662 (0.623, 0.695)	0.614 (0.575, 0.649)
nortriptyline	0.545 (0.503, 0.582)	0.619 (0.579, 0.654)	0.622 (0.582, 0.657)	0.638 (0.597, 0.672)
paroxetine	0.518 (0.483, 0.551)	0.622 (0.589, 0.653)	0.622 (0.589, 0.652)	0.611 (0.578, 0.642)
sertraline	0.530 (0.512, 0.546)	0.625 (0.609, 0.640)	0.627 (0.610, 0.642)	0.625 (0.609, 0.641)
venlafaxine	0.554 (0.525, 0.580)	0.639 (0.612, 0.664)	0.639 (0.612, 0.664)	0.607 (0.580, 0.632)
Avg	0.529 (0.519, 0.537)	0.628 (0.619, 0.636)	0.630 (0.621, 0.638)	0.619 (0.610, 0.627)

Supplemental eTable 6: AUC performance for different feature representations evaluated on the Site B test set. Using *extreme random trees (XRT)* classifiers trained to predict *general* stability.

© 2020 Hughes MC et al. *JAMA Network Open.*

eFigure 7. PPV and NPV Tradeoffs for General Stability Classifiers

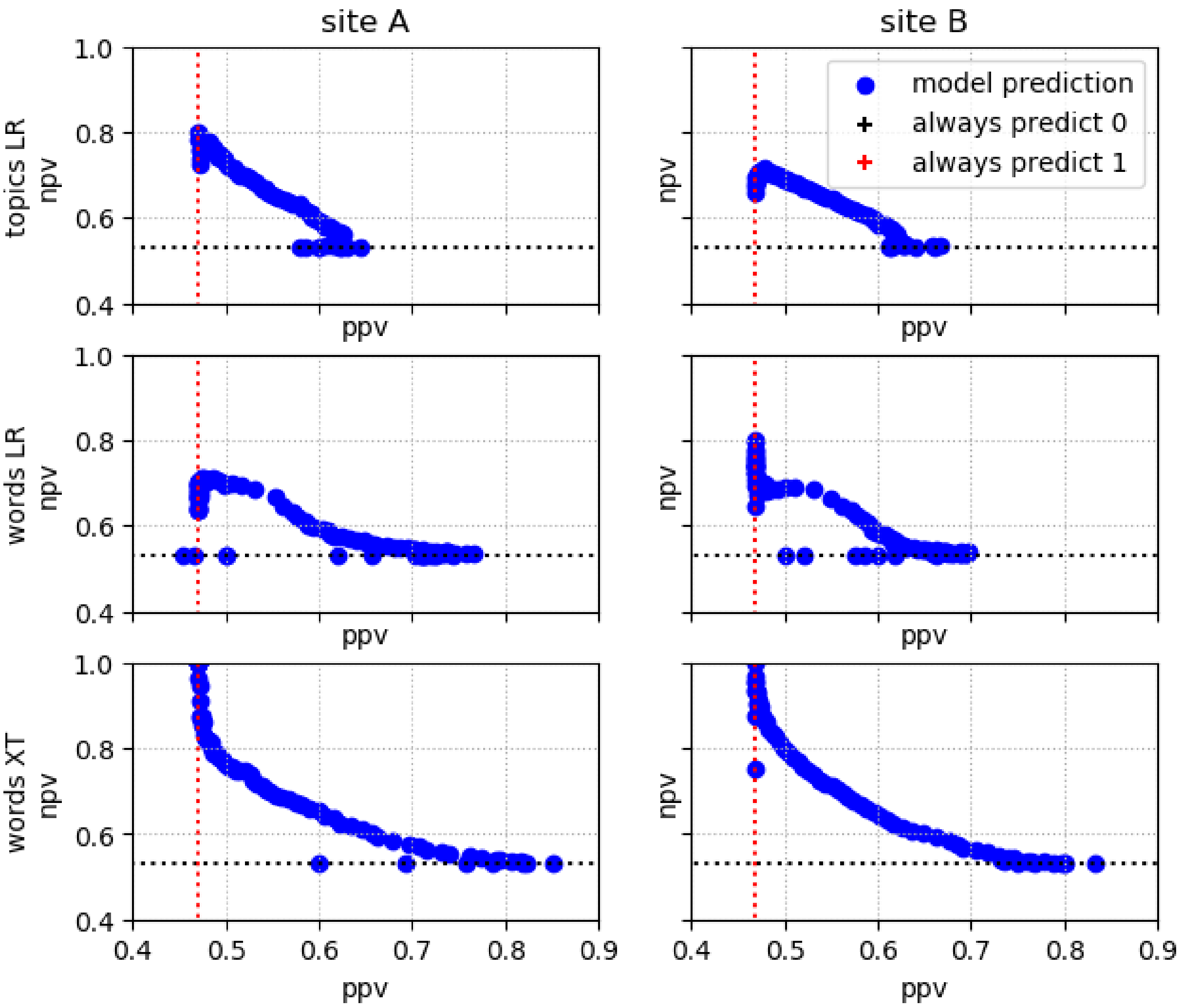

Supplemental eFigure 7: Positive predictive value (PPV, higher is better) and negative predictive value (NPV, higher is better) for the binary classifiers trained to predict general stability, evaluated on Site A and Site B across all subjects. We show logistic regression (LR) and extremely randomized trees (XRT) classifiers across both 10-dimension "topics" and 9256 -dimensional "words" features. Each classifier is trained to produce a real-valued probability score for a given input. In each plot, we sweep across a range of possible decision thresholds for converting scores into binary decisions, to show how classifiers improve on the baselines of always predicting unstable (0) or always predicting stable (1).

© 2020 Hughes MC et al. *JAMA Network Open.*

eFigure 8. Important Features for XRT and LR Classifiers

Extreme Random Trees

0.040	C	v700	routine exam
0.017	d		year of visit
0.013	d		age
0.013	C	99396	preventive exam (age 40-64)
0.013	C	v0481	influenza immunization need
0.011	C	90658	influenza vaccine
0.010	C	80061	cholesterol blood test
0.009	d	99214	office visit >=25min
0.009	d		gender female
0.008	C	99213	office visit >=15min

Logistic Regression

0.028	I	v700	routine exam
0.022	M	8640	prednisone
0.021	M	2231	cephalexin
0.021	M	5640	ibuprofen
0.020	C	99000	lab specimen handling
0.019	C	80061	lipid panel
0.018	M	10831	sulfamethoxazole
0.017	M	46041	alendronate
0.015	M	37418	sumatriptan
0.015	M	591622	varenicline
-0.028	C	90801	psych. interview
-0.022	M	15996	mirtazapine
-0.021	C	80100	drug screen
-0.019	I	3091	prolonged depression
-0.018	M	321988	escitalopram
-0.017	M	42347	bupropion
-0.016	I	v220	first pregnancy
-0.016	C	70450	brain tomography
-0.014	C	93005	electrocardiogram
-0.014	I	e8889	unspecified fall
-0.013	C	99201	office visit >10 min.

Supplemental eFigure 8: Visualization of learned features important to two baseline supervised machine learning predictors of stable treatment in general. We examined logistic regression (LR) and ensembles of extremely randomized decision trees (XRT) using the "dem+words" feature representation, which combines the 9,256-dimensional sparse histogram features of EHR codewords and the simple demographic features of age, gender, race, and year of evaluation visit. *Left*: We show the 10 most informative codewords for our extremely randomized trees ensemble classifier as ranked by Scikit Learn's built-in measure of information theoretic relevance of each individual feature to the overall prediction. Note that these measures are not signed, so each shown feature's presence might be positively or negatively correlated with stability. *Right*: For logistic regression, we show the top 10 terms with most positive weight coefficient (top), as well as the top 10 terms with most negative coefficients (bottom). All features can be categorized as coming from ICD-9/10 codes ("I"), CPT procedural codes ("C"), medication codes ("M"), or demographics ("d"), as indicated in the second column. The third column provides the original code itself.

© 2020 Hughes MC et al. *JAMA Network Open.*

eTable 7. Top-3 Stability Accuracy Comparison of Models With Clinical Practice

Features	Clf.	Top-3 Accuracy	Num. Assessable	Num. Total	Perc. Assessable
topics	LR	0.581 (0.566, 0.594)	4870	12762	38.2%
topics	XRT	0.578 (0.564, 0.590)	5097	12762	39.9%
dem+words	LR	0.591 (0.578, 0.604)	5335	12762	41.8%
dem+words	XRT	0.622 (0.610, 0.634)	6056	12762	47.4%
Common stable meds		0.602 (0.591, 0.612)	8178	12762	64.1%
Observed Rx.		0.602 (0.593, 0.611)	12699	12762	99.5%
Observed Rx. filled up with common stable		0.637 (0.628, 0.646)	12736	12762	99.8%

Supplemental eTable 7: Evaluation of drug-specific stability models as used to prioritize medications for patients. For each subject in the Site A test set, we imagine suggesting a set of 3 personalized antidepressants (the top 3 ranked by each drug's predicted probability of stability). We rank a patient "assessable" if any of these top 3 recommendations are known to be either stable or non-stable. We compute "top 3 accuracy" as the fraction of assessable patients that had at least one of their top 3 stable. We report the accuracy on the Site A test set, as well as in parentheses the 95% confidence interval from 1000 bootstrap samples of the Site A test set. Each row shows a different stability prediction model determined by input features ("dem+words": patient sociodemographics plus codeword count vectors; "topics": our proposed 10-dimensional learned features) and a different classifier ("LR": logistic regression; "XRT": extremely randomized ensemble of 512 decision trees). We also compare to several baselines. "Common stable meds" means always suggesting the same 3 antidepressants most commonly associated with stable treatment in the Site A training set. "Observed Rx." means suggesting exactly the same subset of the 11 target drugs that were prescribed at the index visit in the patient's record. "Observed Rx. filled up with common stable" means that if the record shows less than 3 drugs prescribed, we add to the observe prescriptions the one-size-fits-all "common stable" drugs until 3 total drugs are considered. "Observed Rx." shows a small fraction of subjects (<1%) as not assessable because a few subjects were only prescribed drugs outside the set of our 11 target drugs for which we had enough data to build and test models.

© 2020 Hughes MC et al. *JAMA Network Open.*

eTable 8. Number of Medication Changes Needed by Predicted Stability Quartile

Features	Clf.	Average Num. Med. Changes for 1st quartile	Average Num. Med. Changes for 2nd quartile	Average Num. Med. Changes for 3rd quartile	Average Num. Med. Changes for 4th quartile
topics	LR	1.722 (1.647, 1.799)	1.356 (1.288, 1.428)	1.134 (1.063, 1.204)	0.864 (0.816, 0.918)
topics	XRT	1.721 (1.639, 1.795)	1.359 (1.289, 1.435)	1.119 (1.057, 1.189)	0.878 (0.825, 0.936)
dem+words	LR	1.682 (1.609, 1.771)	1.389 (1.316, 1.465)	1.145 (1.072, 1.213)	0.860 (0.801, 0.924)
dem+words	XRT	1.754 (1.681, 1.843)	1.451 (1.377, 1.528)	1.135 (1.066, 1.207)	0.736 (0.688, 0.796)

Supplemental eTable 8: Average number of additional changes to patient's prescribed antidepressants beyond the first (index) treatment, as observed in the Site A test set, stratifying by quartiles of predicted probability of general stability. 1st quartile corresponds to lowest 25% of test set subjects by probability of stability at index visit; 4th quartile corresponds to the top 25% of test set subjects by probability of stability at index visit. Each row shows a different general stability prediction model, determined by selecting input features ("dem+words": patient sociodemographics plus codeword count vectors; "topics": our proposed 10-dimensional learned features) and a specific classifier ("LR": logistic regression; "XRT": extremely randomized ensemble of 512 decision trees). We report the mean number of additional visits at each quartile and in parentheses the 95% confidence interval for this mean based on 1000 bootstrap samples of the Site A test set.

© 2020 Hughes MC et al. *JAMA Network Open.*

eResults 1. Visualizations of Learned Models

Supervised topic model visualizations

We have created interactive HTML visualizations of the trained topic-word parameters and regression coefficients for all supervised topic models used in this study. Simply point your web browser to the links below to browse.

- PC-sLDA topic model with 10 topics for general stability (model shown in Figure 3)

Https://www.eecs.tufts.edu/~mhughes/research/htmlviz/v20190920/rank_words_by=proba_word_given_topic/MODEL_NAME=general-LABEL_NAME=any-N_STATES=10.html

- PC-sLDA topic model with 10 topics for drug-specific stability with bupropion

Https://www.eecs.tufts.edu/~mhughes/research/htmlviz/v20190920/rank_words_by=proba_word_given_topic/MODEL_NAME=specific-LABEL_NAME=bupropion-N_STATES=10.html

Some of the code words have extremely long descriptions, so we show by default only the first 30 characters. If you hover your mouse over any word that is cut-off, you'll see the entire description.

Baseline classifier visualizations of feature importance

We have also created interactive HTML visualizations of the top ranked features for both the logistic regression and extremely randomized trees (ensemble of 512 decision trees) classifiers used in this study.

- Extremely randomized trees for general stability (also shown in eFigure 8 left)

Https://www.eecs.tufts.edu/~mhughes/research/htmlviz/v20190920/rank_words_by=proba_word_given_topic/MODEL_NAME=general-FEAT_NAME=dem+words-CLF_NAME=extreme_random_trees-LABEL_NAME=any_drug.html

- Logistic regression for general stability (also shown in eFigure 8 right)

Https://www.eecs.tufts.edu/~mhughes/research/htmlviz/v20190920/rank_words_by=proba_word_given_topic/MODEL_NAME=general-FEAT_NAME=dem+words-CLF_NAME=logistic_regr_l2-LABEL_NAME=any_drug.html

- Logistic regression for drug-specific stability with bupropion.

Https://www.eecs.tufts.edu/~mhughes/research/htmlviz/v20190920/rank_words_by=proba_word_given_topic/MODEL_NAME=specific-FEAT_NAME=dem+words-CLF_NAME=logistic_regr_l2-LABEL_NAME=bupropion.html

© 2020 Hughes MC et al. *JAMA Network Open.*

eResults 2. Stability Outcomes for Patients at Site A and Site B

Site A. Overall at Site A (n=53,643), we observed that 16,850 (31%) patients were never stable, 25,141 (47%) of patients reached stability on the index prescription, and 11,652 (22%) reached stability eventually.

For psychiatrist-treated patients (n=11,985 at Site A), we observed that 2,642 (22%) never reached stability, 5,274 (44%) reached stability on the index prescription, and 4,069 (34%) reached stability eventually. In contrast, for primary care patients (n=14208 at Site A), 14,208 (34%) never reached stability, 19,867 (48%) were stable following index prescription, and 7,583 (18%) were eventually stable.

Site B. Overall at Site B (n=27,987), we observed that 9,477 (34%) patients were never stable, 13,018 (47%) of subjects reached stability on the index prescription, and 5,492 (20%) reached stability eventually.

For psychiatrist-treated patients (n=5,267 at Site B), we observed that 1,163 (22%) never reached stability, 2,502 (48%) were stable on the index prescription, and 1,602 (30%) eventually reached stability. In contrast, for non-psychiatrist-treated patients (n=22,720 at Site B), 8,314 (37%) never reached stability, 10,516 (46%) were stable following index prescription, and 3,890 (17%) were eventually stable.

© 2020 Hughes MC et al. *JAMA Network Open.*

eMethods 1. Procedures for Study Design, Outcome Definition, and Prediction Task Formulation

In light of space constraints in the main manuscript, in this supplemental section we provide a more detailed description of our cohort derivation, outcome definition, and formulation as a prediction task.

Our study's goal is to build and evaluate prediction models that can take as input a patient's history (as represented in diagnostic codes, procedural codes, medications, and demographics) and produce predictions of stable antidepressant treatment (either in general or with a specific target drug). To build and assess a stability prediction system from an observational dataset required several carefully considered steps described in the subsections below. First, we defined an initial patient cohort (see "*Initial Cohort Definition*") based on EHR records from our two major hospital systems (Site A and Site B). Next, we identified antidepressant treatments of interest and divided each patient's record into segments of constant prescription (see "*Dividing Patient Record into Segments of Antidepressant Treatment*"). We then developed an outcome defining for each segment whether it was stable or not stable (see "*Determining Stability Outcome for each Segment of Antidepressant Treatment*"). Finally, we formulated the task of stability prediction as a supervised machine learning problem and developed an approach to train and evaluate prediction models. This required for each patient determining specific dates within the observed treatment history at which we want to provide and evaluate stability predictions (see "*Selecting Specific Dates in Patient Record when Predictions are Performed")*. It further required dividing Site A patients into training, validation, and test populations and well as a Site B testing population (see "*Forming Experimental Cohorts for Training and Evaluating Stability Prediction").*

Initial Cohort Definition

Inclusion Criteria. The study cohort included individuals with at least one diagnosis of major depressive disorder (MDD, ICD9 codes 296.2x, 296.3x) or depressive disorder not otherwise specified (ICD9 code 311) who received psychiatric care between December 1 1997, and December 31, 2017, across the inpatient and outpatient networks of two large academic medical centers in New England ("Site A" and "Site B"). Patients were excluded if age was less than 18 or greater than 80, if the total observation period was less than 90 days, or if there were fewer than 3 total documented visits (of any type, psychiatric or otherwise) in the EHR.

Available Data. For each matching individual in the preliminary dataset, we extracted a deidentified patient-specific EHR using the i2b2 server software (i2b2, Boston, MA, USA). Available patient data includes sociodemographic information (age, sex, race/ethnicity), all diagnostic and procedure codes (each associated with a specific visit date), and detailed information about inpatient and outpatient medication prescriptions. Each prescription record indicates the ingredient, the start date, the source (inpatient or outpatient), and (if available) the prescribed number of units per day, total units dispensed, total duration in days, number of refills, and the stop date.

Responsible Data Use. The Partners HealthCare institutional review board approved the study protocol, waiving the requirement for informed consent as only deidentified data was utilized and no human subjects contact was required.

Dividing Patient Record into Segments of Antidepressant Treatment

Active Care Intervals. We define an active care interval for antidepressant treatment as beginning at an index prescription date (i.e. treatment initiation, prior to which there was no antidepressant prescription for at least 13 months (390 days)) and continuing to include all later antidepressant prescriptions until there is a gap of over 390 days during which no prescriptions occur. Given the large number of available observations, we elected to include for each patient only the first observed interval that meets relevant index visit criteria and sufficient follow-up criteria.

27 Possible Drugs Considered for Stability. During an active care interval, several different antidepressants might be prescribed, whether in combination or in sequence. We consider prescriptions for any of 27 antidepressant

medications approved in the United States (see Supplemental eTable 1), selected based on a recent systematic review of antidepressants[1]. Any treatment regimen that includes any of these drugs is rated as stable or not stable.

11 Target Drugs Evaluated for Prediction. To later build and assess drug-specific models of stability, we narrowed focus to only the 11 most commonly-prescribed drugs (see Supplemental eTable 1). This filtering ensures sufficient representation of each treatment in the available data. These 11 target drugs were all prescribed at least once to over 1000 patients in the Site A training set; others were far more infrequent and thus set aside. We only build drug-specific models for these target drugs, and only evaluated on subpopulations that were prescribed one of these drugs. However, we emphasize that the larger set of 27 possible drugs is still considered. For example, a patient in the test set might have first used a target drug, then later been stable on a non-target drug.

Index Visit Criteria. For each patient, we define possible index visits (the start of an active care interval) as visit dates where the patient begins any prescription for one or more of the possible 27 antidepressants. Before this index visit, they must have no recent history of treatment (no recorded prescriptions for any of the 27 antidepressants for at least the previous 390 days). Index visits occurred after April 1, 2008.

Sufficient Follow-up Criteria. To be eligible for inclusion, an active care interval must also have met sufficient follow-up criteria. First, in addition to the index visit, there must have been at least one additional antidepressant prescription (for any of the 27 possible drugs) dated between 30 and 390 days after the index visit. Second, the care interval's total duration (measured by using duration information from the prescription records) must be at least 90 days.

Segments of Antidepressant Treatment. For each patient, we divided the active care interval into segments: contiguous periods where the subject was repeatedly prescribed either one single antidepressant or one consistent combination of antidepressants. Each segment lasts from its initial prescription date until the end of its duration (using prescription durations available in the records).

In summary, for each eligible patient, we included exactly one active care interval that meets all of the following requirements: it includes at least 2 prescriptions events, has total duration of at least 90 days, and contains no gap between prescriptions of longer than 390 days. We evaluated each segment within this active care interval for stability, using the criteria below.

Determining Stability Outcome for each Segment of Antidepressant Treatment

Rationale. Recognizing that traditional clinical trial outcomes such as response and remission are difficult to define reliably for all individuals using solely coded clinical data, we instead sought to identify individuals who achieved a period of stable treatment, as a proxy for ample clinical benefit and tolerability. We applied a simplifying but face-valid assumption that successful treatments continue uninterrupted over time with repeated prescriptions, while unsuccessful treatments are either discontinued or require addition of further medication.

Stability Criteria. Given each patient's eligible active care interval, we divided the record into non-overlapping treatment segments. We then defined a treatment segment as stable if it contained at least two prescriptions for the same antidepressant(s) on two distinct dates, the total duration was at least 90 days, the calculated medication possession ratio (fraction of days in segment where the patient possessed a valid, non-expired prescription)[2] was at least 80%, and the largest gap between the start dates of any two prescriptions in the segment was between 30 days and 390 days. The 30-day minimum between prescription dates ensures that enough time has elapsed for the patient to acclimate to the medication and exhibit all long-term side effects. Any segments within an eligible active care interval that did not meet all stability criteria were considered non-stable.

To illustrate clinical course, example patient trajectories showing various treatment segments over time that satisfy (or do not satisfy) stability criteria are illustrated in Supplemental eFigure 2 and eFigure 3. As expected, a subset of patients never reach stability despite multiple different attempted treatments.

Selecting Specific Dates in Patient Record when Predictions are Performed

Evaluation Dates. To select evaluation dates (events in the historical record where we'd like to make and evaluate predictions), we chose to focus on supporting stability prediction specifically for patient-clinician encounters where the clinician believes a medication change may be required. That is, we would like to be able to make a prediction at each date in the patient record corresponding to a prescribed change in antidepressant treatment. We naturally limited evaluation dates to periods where the patient met active care criteria (defined above), so we could better distinguish stability (or lack thereof) from issues related to patients not receiving any follow-up care.

Ensuring Reliability of EHR Prescription Records. Because electronic prescribing across our two health systems was only mandated starting October 1, 2007, we restricted possible evaluation visits to those occurring after a minimum calendar date of April 1, 2008. This 6-month lead-in enabled treatment that has just begun to be distinguished from treatment continued from the period prior to e-prescribing. This ensures that any event we consider an index prescription is reliable. While this restriction limits the dates at which we evaluate predictions of stable treatment, we emphasize that patient history can still contain features from any time prior to the index prescription, even those before 2008. This should better reflect real-world data.

Evaluation focused on index visits only. To train and evaluate prediction models in this work, we consider only the antidepressant prescription segment corresponding to the index visit (first visit) of the eligible active care interval. We leave evaluation of models that account for multiple segments in series to future work. We did assess stability at both index and later visits, but did not make predictions or evaluate them at later dates.

Forming Experimental Cohorts for Training and Evaluating Stability Prediction

Sufficient history criterion. We further excluded data from individuals who lack sufficient history for prediction - i.e., individuals without any prior diagnostic codes, procedures, or prescriptions (psychiatric or otherwise) at the index visit of the earliest eligible active care interval. We cannot make useful personalized predictions for these subjects, so we exclude them from impacting our evaluation.

Training and testing cohort. After applying all inclusion and sufficient history criteria, a total of 51048 patients from Site A and 26176 patients from Site B were included. The Site A patients were randomly assigned to different subsets labeled training (50%, 25524 patients), validation (25%, 12762 patients), and test (25%, 12762 patients). Using a significant fraction (25% of subjects) for validation and testing ensures better accuracy of held-out performance estimates. Validation and test splits were sampled in a stratified fashion, so that each patient's data belongs in exactly one split and that each target antidepressant's stability outcome label frequency was balanced across splits. Only patients from Site A's training set were used to train any prediction models, while Site B was held-out as an external validation set.

In summary, prediction model training and evaluation included only patients that meet all of our initial inclusion criteria (age 18-80, diagnosis for MDD, at least 3 visits for any reason over at least 90 days), active care eligibility criteria (index antidepressant preceded by 13 months of no antidepressant prescriptions, sufficient follow-up), and sufficient history criteria (at least one code available before index visit). Each such patient from Site A is represented in either the training, validation, or testing set. Each such patient from Site B is represented in that site's testing set. For all such patients, available covariate and outcome data includes the input features and stability outcomes relevant for exactly one evaluation date (the index visit of their active care interval).

© 2020 Hughes MC et al. *JAMA Network Open.*

eMethods 2. Procedures for Classifier Training and Hyperparameter Selection

Here we outline in detail the steps and rationale used for training probabilistic classifiers for general and drug-specific stability, given different possible feature representations (e.g. demographics “dem”, codewords “words”, and “topics”). For scripts necessary to reproduce our analyses, see our public code base online: https://github.com/dtak/prediction-constrained-topic-models/

Outcome Definition and Representation

For training and evaluating stable treatment classifiers, we focused on the index antidepressant treatment segment as the only evaluation date in each patient’s record. Thus, each subject had exactly one feature vector, binary label pair included in evaluation. If the first segment met stability criteria, we associated a positive outcome label with all antidepressants prescribed therein. If the segment was not stable, we associated a negative label for the prescribed antidepressants. Otherwise, if an antidepressant was not used, the associated outcome label was treated as unobserved (stored numerically as not-a-number or “nan”. When training each drug’s specific prediction model, we ignored any subjects which were “unobserved” for that drug. Illustrations of positive, negative, and unobserved (nan) labels were provided in Supplemental Figure 2. We leave the consideration of prediction models for multiple segments in series to future work.

Feature Representations

We considered three possible feature representations of a patient’s history which could be provided as input to personalize predictions at a specified evaluation date X:

“dem”: Sociodemographics and other static features
- Produces a 10-dimensional feature vector concatenating sociodemographics known for all patients
- Includes Patient Gender (one-hot encoded vector of size 3, Male/Female/Undefined)
- Includes Patient Race (one-hot encoded vector of size 5, White/Black/Asian/Hispanic/Other)
- Patient Age at the evaluation date X (as a floating-point numerical value, in units of years)
- Calendar Date of the evaluation date X (as a floating-point numerical value, in units of years since 1970)

“words”: EHR codeword counts of diagnoses, procedures, and prescribed drugs
- Produces a 9256-dimensional count vector of non-negative integers
- Most entries will be exactly zero (sparse).
    - Fraction of non-zero entries: 0.0143 on Site A train and 0.0146 on Site A validation.
- When making predictions at date X, will only include codewords that occur before X

“topics”: Topic membership features summarizing each patient’s EHR codeword counts
- Produces a K-dimensional feature vector of membership probabilities
- Dimension was selected to K=10 for all final experiments. Preliminary experiments considered several possible K values (see eMethods 3: “*Topic Model Training and Hyperparameter Selection*” below)
- Each entry of the feature vector will be a scalar between 0 and 1; the entire vector sums to one
- The k-th entry can be interpreted as “fraction of patient history explained by topic k”
- Computed by performing inference for the latent membership vector given the patient’s “words” vector and a pretrained topic model with K topics. Performs a fast, iterative maximum a-posteriori (MAP) optimization procedure (see Hughes et al.[2] for details)
- When making predictions at date X, will only include codewords that occur before X in computation

Details of topic model training and hyperparameter selection can be found in a later section of this supplement. Here, we focus on simply using the covariates produced using pretrained topic models to make predictions. Our proposed topic models are trained in a supervised fashion. However, we still fit a second stage classifier to these features in the same manner as with baseline features to be sure we have a fair comparison; by using the same protocol for all cases.

© 2020 Hughes MC et al. *JAMA Network Open.*

We also considered several combinations of the feature representations listed here (e.g. “dem+words” or ``dem+topics’’). In these cases, the relevant individual feature vectors were simply concatenated into a longer feature vector.

Classifier Training, Hyperparameter Selection, and Evaluation

As baselines for our binary classification task, we considered two standard probabilistic classifiers, logistic regression (LR) and extremely randomized trees (XRT)[3]. These two baselines were selected because they are representative of two common best-in-class prediction approaches: generalized linear models and random forests. Both methods have been widely used for our data types of interest: tabular data (our “demographics” features) or high- dimensional count data (our “words” features).

Classifier training and model selection details. Using each classifier (LR and XRT), a separate drug-specific classifier for each of the 11 target drugs as well as a general stability classifier (aggregating information from all drugs) was trained on the Site A training set. Hyperparameters were tuned on the Site A validation set, using grid search to find the parameter combination that performed best on the area-under-the-ROC-curve (AUC) discriminative metric. Finally, model performance was compared using AUC for each of the 11 drug prediction tasks in the held-out testing set from Site A, then in the independent Site B.

To indicate uncertainty, we show error bars with 95% confidence intervals of the AUC (i.e. scores at 2.5 and 97.5 percentiles) computed from 5000 bootstrap samples of the test set.

Extremely randomized tree (XRT) details. We used the ensemble.ExtraTreesClassifier (https://scikit-learn.org/stable/modules/generated/sklearn.ensemble.ExtraTreesClassifier.html) implementation available in the Scikit Learn toolkit[4]. We fixed the total number of trees (independent estimators) to 512, which provided a good tradeoff between generalization capability (reduced variance) and computational speed. We then tuned two remaining hyperparameters: the fraction of features used in each tree (max features, possible values = {0.04, 0.16, 0.64}), and the minimum number of samples at leaf nodes (min samples leaf, possible values = {4, 16, 64, 256, 0.1, 0.2, 0.4}). Fractional values for min samples leaf set the leaf size as a fraction of the total training set size. We overcame class imbalance issues by using the provided class weight=‘balanced’ option.

Logistic regression (LR) details. We used the linear model.logisticregression (https://scikit-learn.org/stable/modules/generated/sklearn.linear_model.LogisticRegression.html) implementation in the open-source Scikit Learn toolkit[4]. We elected to control overfitting with an L2 norm penalty on the weight coefficients, but no such penalty on the intercept term). We used the built-in L-BFGS solver (solver=‘lbfgs’), running until the default convergence threshold was achieved (no warnings indicated this threshold was not met). We overcame class imbalance issues by using the provided class weight=‘balanced’ option. The regularization strength hyperparameter “C” (a positive scalar) that controls the L2 penalty was set via grid searched on a logarithmically spaced grid from 10−12 to 106 with 37 possible values.

We emphasize that we use the L2 sum of squares penalty for simplicity. Additional experiments with an alternative sum of absolute values penalty (known as the least absolute shrinkage and selection operator (LASSO)) did not indicate any improved prediction quality. For the dem+words features on the Site A test set, we find L2-regularized logistic regression trained to predict general stability achieves an AUROC averaged across 11 medications of 0.628 (95% bootstrap CI: 0.614 - 0.639), while the L1-regularized method achieves almost the same score of 0.631 (95% bootstrap CI: 0.618 - 0.643). Furthermore, when trained to predict the drug-specific stability, we find that L2-regularized method achieves an average AUROC of 0.627 (95% bootstrap CI: 0.614-0.638), while the L1-regularized method achieves an ever-so-slightly worse test-set score of 0.616 (95% bootstrap CI: 0.603 - 0.627). Because performance differences between L1 and L2 methods differed by at most 0.01 on the AUROC scale, we choose to only report L2-regularized results.

© 2020 Hughes MC et al. *JAMA Network Open.*

eMethods 3. Procedures for Topic Model Training and Hyperparameter Selection

Here we outline in detail the steps and rationale used for training our proposed topic models. For scripts necessary to reproduce our analyses, see our public code base online: https://github.com/dtak/prediction-constrained-topic-models/

Training Duration and Snapshot Selection. All topic modeling methods were allowed to train for 5000 complete passes through the Site A training dataset, or up to 48 hours, whichever came first. Throughout training, each separately initialized training run recorded point estimates of the key parameters (topic-word probabilities and regression coefficients) at regular intervals. After training, we looked across all saved snapshots and selected the single best parameter snapshot according to a score function that balanced discriminative performance (as measured by AUC) and generative performance (as measured by a variational bound on the log likelihood) on the held-out validation set from Site A. The mathematical details of this score function are fully described in our published paper on PC-sLDA[5]. This select-the-best-snapshot-on-validation process can be interpreted as "early stopping," a common machine learning technique to avoid overfitting.

LDA Topic Model Training. As a baseline, we first trained unsupervised Latent Dirichlet Allocation topic models using the collapsed Gibbs sampler implemented in Java via the open-source Mallet toolbox[6]. The relevant hyperparameters specified in advance included the number of topics K (a positive integer), the document-topic Dirichlet prior concentration hyperparameter (a positive scalar), and the topic-word Dirichlet hyperparameter (a positive scalar). We set these parameters via grid search maximizing the score function described above on validation data. For the number of topics, we considered 10, 25, 50, and 100. For the document-topic hyperparameter we tested the scalar values of 0.01 and 0.1, while for the topic-word hyperparameter we tested 0.0033 and 0.0333 (lower values encourage more sparsity). For the LDA Gibbs sampler, at each hyperparameter setting we took the best of 3 separate random initializations, each one using a different random seed and drawing topic-word distribution parameters from a smooth "background" distributions so that no values are too extreme (all words have non-zero probability in all topics) yet each initial topic was distinct enough from others that effective learning could occur. See our public code repository for further details.

PC sLDA Topic Model Training. Our preferred topic modeling approach is the supervised Latent Dirichlet Allocation model fit to data via prediction-constrained (PC) training. This PC-sLDA approach is fully described as a statistical learning algorithm in Hughes et al[5]. Briefly, this method improved over classic unsupervised topic modeling by directly informing the learned topic-word parameters via the intended prediction task (in this case, predict stable antidepressants given patient history). The training process set up an optimization problem for key parameters (topic-word probabilities and logistic regression coefficients) to maximize an objective function that favored both generative performance (as measured by the likelihood of observing EHR codeword count history under the topic model) and discriminative performance (as measured by the log of the conditional probability of binary drug stability outcomes when predicted from the observed patient EHR codeword count history via the topic model).

In addition to the sharing all the concentration hyperparameters of the LDA model, our proposed PC-sLDA method also required specifying a tradeoff scalar Lagrange multiplier λ to upweight the discriminative term in the optimization objective. Following best practices[5], we grid searched for λ values over the fixed possible values of 1, 100, 5000, 10000. For each number of topics K considered (10, 25, 50, and 100), we used the Dirichlet concentration parameters that were selected for the corresponding unsupervised LDA method.

For the stochastic gradient descent (SGD) optimization of PC-sLDA models, we divided all 25,524 patients from the Site A training data into 20 batches of (roughly) equal size. Like the unsupervised topic model, we ran several random initializations from the smooth background distributions. Because the SGD approach is particularly vulnerable to poor exploration of the non-convex energy landscape, we also included additional runs of PC-sLDA which were started at the best-performing parameter snapshots produced by the unsupervised Gibbs sampler at each hyperparameter configuration (number of topics K, document-topic hyperparameter, etc.). We found that this from-best-Gibbs-run initialization yielded the best performance on validation data according to our hybrid discriminative-generative score function, so all results used this initialization.

© 2020 Hughes MC et al. *JAMA Network Open.*

We show results for K=10 topics throughout, because using more topics did not visibly improve heldout predictions.

End-to-end Training of PC-sLDA. When training all topic models, including the topic model illustrated in the main manuscript's Figure 3, we train the topic model (estimating the parameters of the topics) as well as a predictor that uses the topics (estimating the logistic regression weight coefficients on each topic feature) jointly via a gradient-descent optimization procedure. This can be considered "one-stage" or "end-to-end" training.

After end-to-end supervised training, we can then additionally train a (non-linear) classifier. This leads to a "two-stage" approach, where stage one trains topics with a generalized linear model for supervision. Stage two then fixes the resulting features and trains any classifier such as LR or XRT given those features. We report "two-stage" performance numbers in practice throughout the manuscript (e.g. in Figure 1 or Figure 2), because in these cases we can be sure the classifier training and hyperparameter selection are done consistently and fairly whenever we compare "topics" features with "words" features. Overall the computational cost of this second stage is quite affordable (because there are very few topic covariates).

We emphasize that it would be possible to perform "one stage" or "end-to-end" training with any other non-linear classifier that can be trained via gradient descent (e.g. any neural network). Ensembles of decision trees (such as our XRT classifier) are trained via greedy combinatorial optimization algorithms (not gradient descent), so developing one-stage methods for these would require research effort. However, for our proposed approach, we focus on linear predictors because this forces the topics themselves to be directly interpretable as features that are monotonically related with predictions. Given our focus on interpretability, we would rather invest in training better topics (better feature extractors) than in more complicated predictions given topic features.

© 2020 Hughes MC et al. *JAMA Network Open.*

eReferences.

1. Cipriani A, Furukawa TA, Salanti G, et al. Comparative Efficacy and Acceptability of 21 Antidepressant Drugs for the Acute Treatment of Adults with Major Depressive Disorder: A Systematic Review and Network Meta-analysis. Lancet. 2018; 391(10128):1357-1366.

2. Raebel MA, Schmittdiel J, Karter AJ, et al. Standardizing Terminology and Definitions of Medication Adherence and Persistence in Research employing Electronic Databases. Med Care. 2013;51(8 0 3).

3. Geurts P, Ernst D, Wehenkel L. Extremely randomized trees. Mach Learn. 2006; 63(1):3-42.

4. Pedregosa F, Veroquaux G, Gramfort A, et al. Scikit-learn: Machine Learning in Python. J Mach Learn Res. 2011; 12:2825-2830.

5. Hughes MC, Hope G, Weiner L, et al. Semi-Supervised Prediction-Constrained Topic Models. Proc 21st Int Conf Artif Intell Stat AISTATS. 2018.

6.McCallum AK. MALLET: Machine Learning for Language Toolkit. http://mallet.cs.umass.edu. 2002.

© 2020 Hughes MC et al. *JAMA Network Open.*
